# Development and internal validation of a new Varicose Vein Recurrence Score for open superficial venous surgery

**DOI:** 10.1016/j.jvsv.2026.102583

**Published:** 2026-07-24

**Authors:** Dragan Nikolić, Katarina Petrović, Vladimir Manojlović, Slavko Budinski, Nikola Batinić, Marijana Basta Nikolić

**Affiliations:** a Faculty of Medicine, University of Novi Sad, Novi Sad, Serbia; b Clinic for Vascular and Endovascular Surgery, University Clinical Center of Vojvodina, Novi Sad, Serbia; c Center for Radiology, University Clinical Center of Vojvodina, Novi Sad, Serbia

**Keywords:** Duplex ultrasound, Open venous surgery, Predictive model, Recurrence, Risk score, Varicose veins

## Abstract

**Background:**

Recurrence after open superficial venous surgery occurs in 20% to 60% of patients at 5 years, yet no validated composite preoperative scoring system exists to quantify individual risk. We aimed to develop and internally validate a predictive model and a derived clinical risk score—the Varicose Vein Recurrence (VVR) Score—for 5-year recurrence after open surgery of the superficial venous system.

**Methods:**

This retrospective single-center cohort study included consecutive patients undergoing saphenofemoral or saphenopopliteal junction ligation with or without trunk stripping and concomitant phlebectomies between January 2006 and December 2025. The primary outcome was duplex-confirmed recurrent reflux with clinically visible recurrent varices at 5 years; reintervention (open, endovenous, or foam) within 5 years served as a prespecified harder secondary end point. The primary analysis was conducted on extremities with complete 5-year outcome ascertainment. We used multivariable logistic regression with cluster-robust standard errors (patient-level clustering) for model development and cluster bootstrap resampling (500 replications) for internal validation. A simplified integer-based score was derived from shrinkage-adjusted coefficients. Prespecified sensitivity analyses included procedure-stratified subgroup models (three mutually exclusive primary subgroups—high ligation + stripping, high ligation alone, and phlebectomy alone—plus an overlapping saphenopopliteal junction-procedure subgroup), continuous calendar-year analyses with time × predictor interactions, treatment choice as a model covariate, inverse probability of censoring weighting, and Cox regression. Reporting followed the Transparent Reporting of a Multivariable Prediction Model for Individual Prognosis or Diagnosis guidelines (type 1b).

**Results:**

Among 12,480 eligible extremities (9210 patients), 10,142 (81.3%) had complete 5-year ascertainment and constituted the primary cohort. The 5-year recurrence rate was 25.1% (2546/10,142); 74.1% of recurrent extremities underwent reintervention (5-year reintervention rate, 18.6%). Six preoperative predictors were retained: anterior saphenous vein (formerly anterior accessory saphenous vein) reflux (odds ratio [OR], 2.33; 95% confidence interval [CI], 2.03-2.67), incompetent perforators (OR, 2.04; 95% CI, 1.77-2.35), body mass index of ≥30 kg/m^2^ (OR, 1.82; 95% CI, 1.60-2.07), deep venous reflux or obstruction (OR, 1.72; 95% CI, 1.44-2.06), Clinical, Etiologic, Anatomic, and Pathophysiologic classification C4-C6 (OR, 1.62; 95% CI, 1.42-1.86), and great saphenous vein diameter ≥7 mm (OR, 1.41; 95% CI, 1.23-1.62). The optimism-corrected C-statistic was 0.744 (95% CI, 0.726-0.762), with a calibration slope of 0.97 (0.924-1.016) and an intercept of −0.02 (−0.06 to 0.02). Model performance was preserved for the harder reintervention end point (C-statistic, 0.73) and across operative subgroups (apparent C-statistics, 0.70-0.74). The derived VVR Score (0-13 points) stratified patients into low (12.8%), moderate (25.2%), high (41.7%), and very high (66.8%) 5-year recurrence-risk categories. Continuous calendar-year analysis showed no significant time × predictor interactions (smallest *P* = .21). Sensitivity analyses including inverse probability of censoring weighting on all 12,480 extremities, Cox regression, generalized estimating equations, and treatment-choice-adjusted models produced consistent results.

**Conclusions:**

The VVR Score provides an internally validated tool for estimating 5-year recurrence risk after open superficial venous surgery using six preoperative clinical and duplex parameters. Performance was preserved across operative subgroups and for the harder reintervention end point. Because the score is derived in an open-surgical population, external validation in geographically diverse open-surgical cohorts and rederivation/recalibration before use in endothermal, nonthermal, or foam-sclerotherapy populations are essential before broader clinical adoption.

**Article Highlights**

• **Type of Research:** Retrospective single-center cohort study (2006-2025) for prediction-model development with internal validation; reporting follows Transparent Reporting of a Multivariable Prediction Model for Individual Prognosis or Diagnosis (type 1b)
• **Key Findings:** Among 10,142 operated extremities, six preoperative variables—anterior saphenous vein reflux; incompetent perforators; body mass index ≥30 kg/m^2^; deep venous reflux/obstruction; Clinical, Etiologic, Anatomic, and Pathophysiologic classification C4-C6; and great saphenous vein diameter ≥7 mm—independently predicted 5-year recurrence. The integer-based Varicose Vein Recurrence Score (0-13 points) achieved an optimism-corrected C-statistic of 0.744 with excellent calibration (slope, 0.97; intercept, −0.02) and stratified patients into four risk categories with observed 5-year recurrence rates of 12.8%, 25.2%, 41.7%, and 66.8%.
• **Take Home Message:** The Varicose Vein Recurrence Score is, to our knowledge, the first internally validated, integer-based preoperative tool for predicting 5-year recurrence after open superficial venous surgery, suitable for structured patient counseling and risk-stratified follow-up after external validation.

Chronic venous insufficiency affects up to 40% of Western adults, with varicose veins as its most common manifestation.^1^^,^^2^ Open surgery—saphenofemoral junction (SFJ) or saphenopopliteal junction (SPJ) ligation with or without trunk stripping and phlebectomies—has been the historical standard, displaced in many high-resource systems by endothermal ablation, nonthermal/nontumescent ablation, and foam sclerotherapy^3^^,^^4^ but still routinely performed in regional vascular centers for recurrent disease, very tortuous trunks, and limited endovenous resources. The Varicose Vein Recurrence (VVR) Score is intended for use in open-surgical populations.

Despite operative refinements, recurrence remains common (5-year rates, 20%-60%).^5^, ^6^, ^7^, ^8^ In our institutional experience, roughly one in four operated extremities develops clinically relevant recurrence, and one in five requires reintervention within 5 years. Mechanisms—neovascularization at the SFJ/SPJ,^9^, ^10^, ^11^, ^12^ residual reflux in treated trunks including stripped-trunk recanalization,^10^ disease progression with new reflux pathways,^13^ and new incompetent perforators—are categorized within the Recurrent Varices After Surgery framework.^6^^,^^14^ Up to three-quarters of patients with recurrence eventually undergo reintervention,^15^^,^^16^ and several individual risk factors are recognized (obesity, advanced Clinical, Etiologic, Anatomic, and Pathophysiologic classification (CEAP), perforator incompetence, and deep venous pathology),^8^^,^^13^^,^^17^, ^18^, ^19^ but no validated composite preoperative score integrating clinical stage with comprehensive duplex anatomy (anterior saphenous vein [ASV], perforators, and deep system) is currently available for open-surgery use.^20^^,^^21^

We therefore aimed to develop and internally validate a multivariable predictive model and a derived clinical scoring system—the VVR Score—for 5-year recurrence after open superficial venous surgery, following Transparent Reporting of a Multivariable Prediction Model for Individual Prognosis or Diagnosis guidance (type 1b: development with internal validation).^22^^,^^23^

## Methods

### Study design and setting

This retrospective cohort study was conducted at the Clinic for Vascular and Endovascular Surgery, University Clinical Center of Vojvodina, Novi Sad, Serbia, within a regional health care system serving approximately 2,000,000 inhabitants. The study spanned 20 years (January 1, 2006 to December 31, 2025). The protocol was approved by the Ethics Committee of the University Clinical Center of Vojvodina (approval number: EC-2024/0847), and informed consent was waived, given the retrospective, anonymized design. The study adhered to the Declaration of Helsinki.^24^

### Study population

The study population comprised consecutive patients who underwent elective open surgery of the superficial venous system during the study period. Eligible procedures comprised two nonoverlapping groups: (1) SFJ and/or SPJ ligation (hereafter termed “high ligation” [HL]), with or without saphenous trunk stripping and concomitant phlebectomies; and (2) ambulatory phlebectomy alone (Müller technique through 1-3 mm stab incisions) for symptomatic varicose tributaries in extremities without truncal reflux on preoperative duplex. The unit of analysis was the operated extremity; in patients with bilateral procedures, each extremity was analyzed separately with statistical correction for within-patient clustering (see Statistical analysis section). For extremities with more than one procedure during the study period, only the first eligible (index) operation was included.

**Fig 1:**
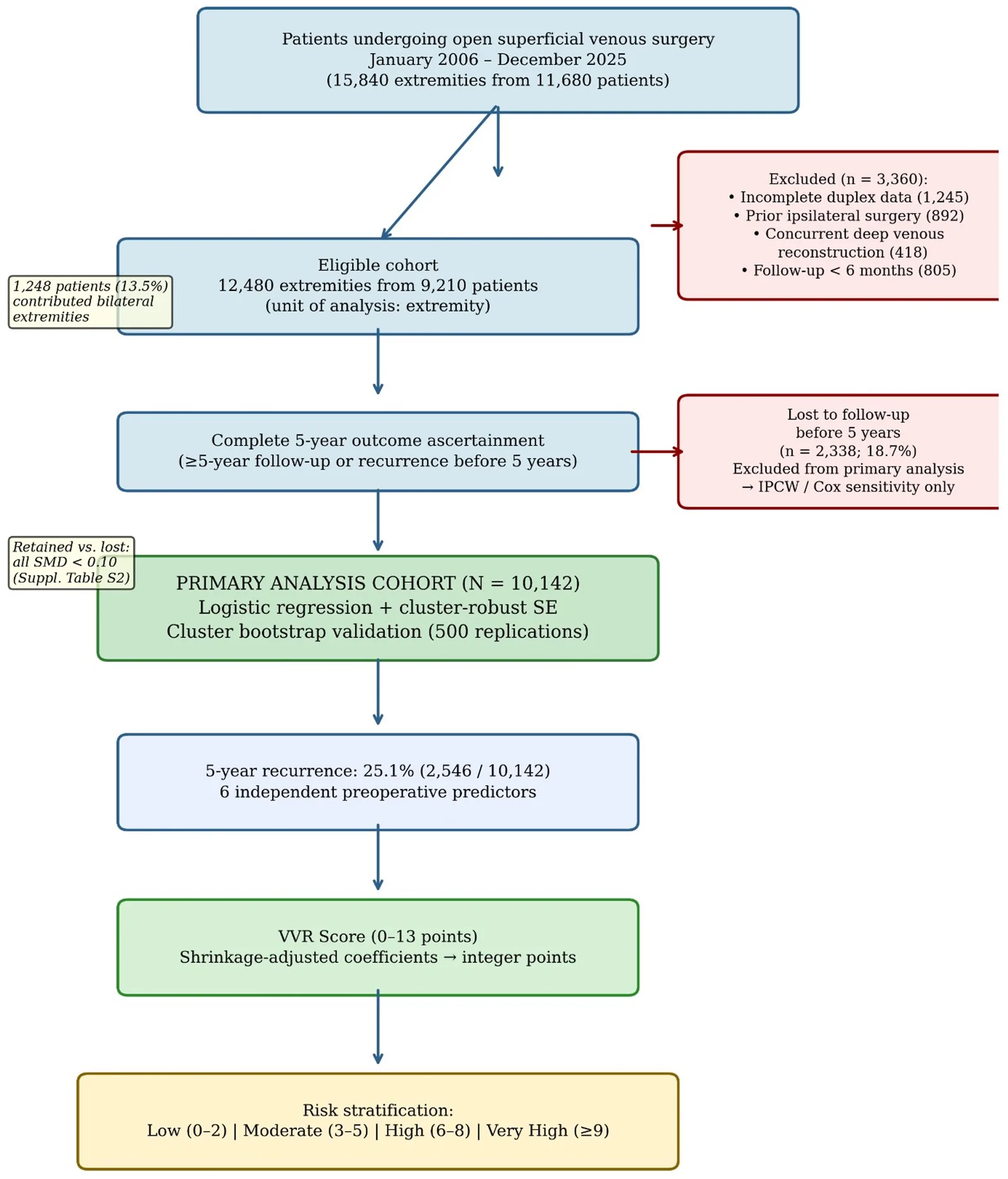
Patient flow diagram. The unit of analysis is the extremity; patients may contribute up to two extremities. The primary analysis cohort comprised 10,142 extremities with complete 5-year outcome ascertainment. Extremities lost to follow-up (n = 2338) were included in IPCW and Cox regression sensitivity analyses on the full eligible cohort (n = 12,480). *IPCW*, Inverse probability of censoring weighting; *SE*, standard error; *SMD*, standardized mean difference; *VVR*, Varicose Vein Recurrence.

Inclusion criteria were (1) open surgical procedure on the superficial venous system as defined above; (2) availability of a standardized preoperative duplex ultrasound examination; and (3) minimum postoperative follow-up of 6 months or documented recurrence within this period. Exclusion criteria were (1) incomplete preoperative duplex data for key anatomical variables (n = 1245); (2) previous ipsilateral venous surgery (n = 892); (3) concurrent reconstructive surgery of the deep venous system (n = 418); and (4) follow-up less than 6 months without documented recurrence (n = 805). Patient flow is detailed in Fig 1.

### Indications for surgery

Referral for open surgical treatment followed a combined clinical-anatomical assessment consistent with institutional practice and contemporary consensus.^4^^,^^25^ All operated extremities had (1) documented chronic venous disease symptoms and/or signs, including limb pain or aching, heaviness or fatigue, ankle edema, night cramps, itching, skin changes (pigmentation, eczema, and lipodermatosclerosis), or healed/active venous ulceration; (2) CEAP clinical class C2 (with venous symptoms) through C6; and (3) duplex-confirmed pathological superficial reflux (>0.5 seconds in the standing position) involving the great saphenous vein (GSV), small saphenous vein, or ASV in extremities undergoing junctional or truncal surgery, per the Union Internationale de Phlébologie (International Union of Phlebology)/European Society for Vascular Surgery consensus^26^^,^^27^; extremities undergoing isolated ambulatory phlebectomy had symptomatic varicose tributaries without truncal reflux on preoperative duplex. The threshold for deep venous reflux, when reported, was >1.0 seconds. Isolated telangiectasias or reticular veins (CEAP C1) were not considered surgical indications.

### Surgical protocol

Open surgery was performed by a stable team of attending vascular surgeons under spinal or general anesthesia. The institutional protocol—flush SFJ ligation with separate tributary ligation; inverted stripping of refluxing GSV to just below the knee in 95.7% of stripped extremities; refluxing-ASV management by separate ostial ligation, additional stripping, or phlebectomy of dependent tributaries; partial-trunk small saphenous vein stripping (31.7%) or flush SPJ ligation alone (68.3%); subfascial ligation of pathological (≥3.5 mm, reflux >0.5 seconds) medial calf perforators undertaken only in advanced CEAP classes (C4-C6), consistent with contemporary guideline recommendations^25^; and routine cribriform fascia closure adopted from January 2013 onward—is summarized with era-stratified parameters in Supplementary Table I (online only). Phlebectomy of varicose tributaries was performed through 1- to 2-mm stab incisions (Müller technique).

### Outcome definition

The primary outcome was 5-year VVR, defined by (1) clinically visible recurrent varicose veins; (2) duplex-confirmed pathological reflux (>0.5 seconds) in the treated segment, a new reflux pathway (ASV reflux, recurrent perforators, and neovascularization), or recanalization of a stripped trunk; and (3) a documented decision that retreatment was indicated. Because criterion (3) is influenced by local follow-up intensity, reintervention within 5 years was prespecified as a secondary, harder end point and reported throughout. Isolated telangiectasias or reticular veins without truncal reflux were not classified as recurrence. Recurrence mechanism was classified using the Recurrent Varices After Surgery framework^6^^,^^14^ as (1) progression in new territories; (2) neovascularization at the SFJ/SPJ; (3) treated-segment recurrence (subclassified as stripped-trunk recanalization vs residual GSV stump or cribriform-fascia tributary reflux); or (4) perforator mediated. Reinterventions were captured, regardless of modality and disaggregated by era.

### Candidate predictors and duplex protocol

Candidate predictors, defined a priori without univariable selection or stepwise procedures, included age, sex, body mass index (BMI), diabetes, hypertension, smoking, CEAP class,^28^ GSV diameter (measured 3-5 cm distal to SFJ in standing position), ASV reflux, perforator incompetence (≥3.5 mm with reverse flow >0.5 seconds),^29^ and deep venous reflux/obstruction including post-thrombotic changes. All preoperative duplex examinations were performed in the standing position with linear-array transducers (7-12 MHz); pathological reflux was retrograde flow >0.5 seconds (superficial/perforator) or >1.0 seconds (deep), per the Union Internationale de Phlébologie (International Union of Phlebology)/European Society for Vascular Surgery consensus.^26^^,^^27^ Where ASV reflux was present (n = 2312), mean ASV diameter was 4.6 ± 1.1 mm and 1758 (76.0%) had ASV-dependent superficial varicosities. Post-thrombotic changes were defined by intraluminal echogenic material with irregular wall thickening, noncompressibility without acute thrombus, loss of respiratory phasic flow, or deep reflux >1.0 seconds in a vein with prior-thrombosis morphology. Surveillance comprised clinical visits at 1, 6, and 12 months and annually thereafter, with structured duplex at 6 and 12 months for all patients; after the first year, duplex was performed when recurrence was clinically suspected and mandatorily before any reintervention.

### Missing data and sample size

All six core predictors were complete in 94.9% of extremities (Supplementary Table II, online only); complete-case analysis was primary, with multivariable imputation by chained equation (MICE) (m = 20) as a sensitivity analysis.^30^ With 2546 events and 6 predictors, events-per-variable was approximately 424, well above thresholds of 10 to 20.^31^^,^^32^

### Statistical analysis

The primary model used multivariable logistic regression on extremities with complete 5-year ascertainment (n = 10,142) with cluster-robust standard errors at the patient level (3270 of 9210 patients [35.5%] had bilateral procedures).^33^ Extremities censored before 5 years (n = 2338; 18.7%) were excluded from the primary model and addressed in inverse probability of censoring weighting (IPCW) and Cox sensitivity analyses; baseline characteristics were comparable between retained and censored groups (all standardized mean differences <0.10; Supplementary Table III, online only). All candidate predictors (age, sex, BMI, diabetes, hypertension, smoking, CEAP class, GSV diameter, ASV reflux, perforator incompetence, and deep venous reflux/obstruction) were entered together; univariable analyses were descriptive and not used for selection. Variables with |β| < .10 and *P* > .15 whose removal did not alter retained coefficients (≤3% Δβ) or discrimination (Δarea under the curve ≤0.005) were not retained in the parsimonious model used to derive the integer score (full coefficients are provided in Supplementary Table IV, online only).^34^ Continuous predictors were tested for nonlinearity with restricted cubic splines and dichotomized at established clinical thresholds (BMI, 30 kg/m^2^; GSV, 7 mm) for the score; multicollinearity was assessed with the variance inflation factor.

Three mutually exclusive primary subgroups (HL + stripping, n = 7099; HL alone, n = 1521; and phlebectomy alone, n = 1522) were analyzed in prespecified procedure-stratified models, with an additional overlapping analysis of any SPJ procedure (n = 811; Supplementary Table V, online only). Calendar year was modeled both categorically (era 1: 2006-2012; 2: 2013-2018; and 3: 2019-2025) and continuously (centered at 2015) with time × predictor interaction terms (Supplementary Table VI, online only). Discrimination was assessed by C-statistic and discrimination slope^35^^,^^36^; calibration by plots, intercept, slope, and Brier score^37^; and clinical utility by decision-curve analysis.^38^^,^^39^ Internal validation used patient-level cluster bootstrap (500 replications); a uniform van Houwelingen-Le Cessie shrinkage factor was applied to all coefficients before integer-score derivation.^40^^,^^41^ Predicted 5-year recurrence: *P* = 1/(1 + exp [−linear predictor (LP)]), LP = β_0_ + Σβᵢxᵢ; coefficients in Supplementary Table IV (online only).

Sensitivity analyses comprised (1) follow-up bias (IPCW, Cox); (2) specification (recurrences <12 months excluded, reintervention as hard outcome, generalized estimating equation, MICE imputation (m = 20), and treatment choice as covariate); and (3) subgroup and time analyses. All analyses were performed in R 4.3.2 (rms, pROC, rmda, mice, survival, and ipw).

## Results

### Study population

During the 20-year study period, 15,840 extremities in 11,680 patients underwent open superficial venous surgery. After applying exclusion criteria (Fig 1), 12,480 extremities from 9210 patients were eligible, of whom 3270 (35.5%) contributed bilateral procedures (6540 extremities) and 5940 (64.5%) contributed unilateral procedures (5940 extremities); the resulting total of 12,480 extremities is internally consistent (3270 × 2 + 5940 = 12,480).

Of the 12,480 eligible extremities, 10,142 (81.3%) had complete 5-year outcome ascertainment and constituted the primary analysis cohort; the remaining 2338 (18.7%) were lost to follow-up before the 5-year landmark. Baseline characteristics did not differ meaningfully between retained and lost extremities (Supplementary Table III, online only; all standardized mean differences <0.10). The median follow-up in the primary cohort was 7.1 years (interquartile range, 4.2-11.4).

Baseline characteristics stratified by recurrence status are presented in Table I. The mean age was 53.4 ± 11.8 years, 62.9% were female, and the mean BMI was 28.3 ± 4.7 kg/m^2^. Advanced disease (CEAP C4-C6) was present in 23.3% of extremities. On preoperative duplex, mean GSV diameter was 7.1 ± 1.5 mm, ASV reflux was present in 22.8% (with mean ASV diameter 4.6 ± 1.1 mm and ASV-dependent ventrolateral varicosities documented in 76.0% of refluxing ASVs), incompetent perforators in 28.0%, and deep venous reflux or obstruction in 12.1%.

**Table I:** Baseline characteristics of the primary analysis cohort (N = 10,142)

Variable	Total (N = 10,142)	No recurrence (N = 7596)	Recurrence (N = 2546)	SMD
Demographics				
Age, years (mean ± SD)	53.4 ± 11.8	53.0 ± 12.0	54.6 ± 11.1	0.14
Female sex, n (%)	6382 (62.9)	4832 (63.6)	1550 (60.9)	0.06
BMI, kg/m^2^ (mean ± SD)	28.3 ± 4.7	27.7 ± 4.5	30.2 ± 4.9	0.53
BMI ≥30, n (%)	3568 (35.2)	2286 (30.1)	1282 (50.3)	0.42
Comorbidities				
Diabetes mellitus, n (%)	1015 (10.0)	726 (9.6)	289 (11.3)	0.06
Hypertension, n (%)	3651 (36.0)	2659 (35.0)	992 (39.0)	0.08
Current smoking, n (%)	2130 (21.0)	1561 (20.6)	569 (22.3)	0.04
Clinical classification				
CEAP C2-C3, n (%)	7778 (76.7)	6103 (80.3)	1675 (65.8)	0.33
CEAP C4-C6, n (%)	2364 (23.3)	1493 (19.7)	871 (34.2)	0.33
Duplex ultrasound findings				
GSV diameter, mm (mean ± SD)	7.1 ± 1.5	6.8 ± 1.4	7.9 ± 1.6	0.73
GSV ≥7 mm, n (%)	5243 (51.7)	3645 (48.0)	1598 (62.8)	0.30
ASV reflux, n (%)^a^	2312 (22.8)	1318 (17.3)	994 (39.0)	0.49
Incompetent perforators, n (%)	2840 (28.0)	1706 (22.5)	1134 (44.5)	0.48
Deep venous reflux/obstruction, n (%)	1227 (12.1)	786 (10.3)	441 (17.3)	0.20
Procedure type (mutually exclusive primary categories)				
HL + stripping, n (%)	7099 (70.0)	5344 (70.4)	1755 (68.9)	0.03
HL only, n (%)	1521 (15.0)	1117 (14.7)	404 (15.9)	0.03
Phlebectomy alone, n (%)	1522 (15.0)	1135 (14.9)	387 (15.2)	0.01
Co-occurring secondary procedure (overlapping)				
Any SPJ procedure^a^, n (%)	811 (8.0)	602 (7.9)	209 (8.2)	0.01
Follow-up				
Duration, years (median, interquartile range)^b^	7.1 (4.2-11.4)	7.4 (4.5-11.6)	5.9 (2.8-9.8)	–

*ASV*, Anterior saphenous vein; *BMI*, body mass index; *CEAP*, Clinical, Etiologic, Anatomic, and Pathophysiologic classification; *GSV*, great saphenous vein; *HL*, high ligation; *SD*, standard deviation; *SMD*, standardized mean difference; *SPJ*, saphenopopliteal junction.

Values >0.10 indicate meaningful imbalance.

Baseline comparisons are presented descriptively; SMDs are not used for predictor selection (Transparent Reporting of a Multivariable Prediction Model for Individual Prognosis or Diagnosis item 13b).

Primary analysis cohort: extremities with ≥5-year follow-up or documented recurrence within 5 years. The three primary procedure-type categories (HL + stripping; HL only; and phlebectomy alone) are mutually exclusive and sum to 10,142 (100.0%) of the cohort. The phlebectomy-alone primary category (n = 1522) includes 1472 with phlebectomy only and 50 with concurrent isolated SPJ procedure (ie, SPJ ligation without saphenofemoral junction [SFJ] surgery). SPJ procedures (n = 811, 8.0% of cohort) are reported as a co-occurring secondary procedure that overlaps with the primary categories: 671 (82.7%) co-occurred with HL + stripping for combined SFJ + SPJ disease, 90 (11.1%) co-occurred with HL alone, and 50 (6.2%) were isolated SPJ procedures with concurrent phlebectomy without SFJ surgery (the 50 cases counted within phlebectomy alone as their primary category). SPJ rows are therefore not added to the primary-category subtotals.

a ASV = anterior saphenous vein (formerly anterior accessory saphenous vein, AASV); terminology updated to align with current consensus nomenclature.

b Follow-up in the recurrence group is necessarily shorter because recurrence events truncate follow-up time.

### Recurrence rates and temporal trends

The overall 5-year recurrence rate in the primary cohort was 25.1% (2546/10,142). Cumulative recurrence rates at 1, 3, and 5 years were 5.4%, 15.2%, and 25.1%. Recurrence rates by calendar era were era 1 (2006-2012), 26.8%; era 2 (2013-2018), 24.5%; and era 3 (2019-2025), 23.1%. Continuous calendar-year analysis (Supplementary Table VI, online only) confirmed a small nonsignificant decline (per-year odds ratio [OR] for recurrence, 0.99; 95% confidence interval [CI], 0.98-1.00; *P* = .11), with no significant time × predictor interactions (smallest *P* = .21 for ASV reflux × calendar-year) and no significant nonlinearity (overall nonlinearity, *P* = .31). The era-3 figure should be interpreted with caution because of the inherently shorter maximum follow-up window in this group; the IPCW analysis on the full cohort addresses this concern.

Of the 2546 recurrent extremities, 1886 (74.1%) underwent reintervention within 5 years, yielding a 5-year reintervention rate of 18.6%. The reintervention modality mix was open redo surgery 1158 (61.4%); endothermal ablation 412 (21.8%); ultrasound-guided foam sclerotherapy 296 (15.7%); and mixed/other 20 (1.1%). The proportion of open reinterventions declined across calendar eras (era 1, 82.6% open; era 3, 41.3% open) as endovenous techniques became locally available. The VVR Score performed similarly for the harder reintervention end point, regardless of modality (C-statistic, 0.73; Supplementary Table VII, online only).

Among recurrent cases with documented mechanism (n = 2245; 88.2%), disease progression in new territories accounted for 38.1% (855), neovascularization for 28.5% (640), recurrent reflux in the treated segment for 21.8% (489—of which 332 [14.8% of mechanism-classified recurrences] represented stripped-GSV recanalization and 157 [7.0%] residual GSV stump or cribriform-fascia tributary reflux), and perforator-mediated recurrence for 11.6% (261). Mechanism-specific predictor profiles are presented in Supplementary Table VIII (online only). A separate category of “treatment failure”—lack of clinical improvement without documented duplex pathology—comprised 142 cases (1.4% of operated extremities) over 20 years and is reported but not included in the recurrence count.

### Univariable analysis

Univariable logistic regression results are presented in Table II. The strongest univariable predictors were ASV reflux (OR, 3.22), incompetent perforators (OR, 2.88), BMI ≥30 (OR, 2.42), and CEAP C4-C6 (OR, 2.09). Neither procedure type nor concomitant perforator treatment was significantly associated with recurrence.

**Table II:** Univariable logistic regression analysis for 5-year recurrence (N = 10,142)

Predictor	OR	95% CI	*P* value	Recurrence rate by level	Reference
Age (per 10-year increase)	1.08	1.01-1.16	.021	–	Continuous
Female sex	0.90	0.82-0.99	.030	F 24.3% vs M 26.3%	Male
BMI ≥30	2.42	2.20-2.66	<.001	≥30: 35.9% vs <30: 19.1%	BMI <30
BMI (per 5 kg/m^2^)	1.43	1.33-1.54	<.001	–	Continuous
Diabetes mellitus	1.21	1.05-1.40	.009	DM 28.5% vs no 24.7%	No DM
Hypertension	1.19	1.08-1.31	<.001	HTN 27.2% vs no 23.9%	No HTN
Current smoking	1.11	0.99-1.23	.062	–	Nonsmoker
CEAP C4-C6	2.09	1.90-2.30	<.001	C4-C6: 36.8% vs C2-C3: 21.5%	CEAP C2-C3
GSV ≥7 mm	1.84	1.68-2.02	<.001	≥7: 30.5% vs <7: 19.3%	GSV <7 mm
GSV diameter (per mm)	1.35	1.30-1.40	<.001	–	Continuous
ASV reflux^a^	3.22	2.94-3.53	<.001	Yes 43.0% vs no 19.8%	No ASV reflux
Incompetent perforators	2.88	2.64-3.15	<.001	Yes 39.9% vs no 19.2%	No incompetent perforators
Deep reflux/obstruction	1.83	1.62-2.08	<.001	Yes 35.9% vs no 23.6%	No deep reflux
HL + stripping	0.93	0.84-1.02	.12	–	Other procedures
Concomitant perforator Tx	0.99	0.88-1.12	.89	–	No treatment

*ASV*, Anterior saphenous vein (formerly anterior accessory saphenous vein [AASV]); *BMI*, body mass index; *CEAP*, Clinical, Etiologic, Anatomic, and Pathophysiologic classification; *CI*, confidence interval; *DM*, diabetes mellitus; *F*, female; *GSV*, great saphenous vein; *HL*, high ligation; *HTN*, hypertension; *M,* male; *OR*, odds ratio.

Univariable ORs were estimated using standard logistic regression; cluster-robust standard errors (patient level) were applied only in the multivariable model (Table III). The impact of clustering on univariable estimates was negligible (standard errors within 1%-2%).

a ASV = anterior saphenous vein (formerly anterior accessory saphenous vein, AASV), terminology updated to align with current consensus nomenclature.

### Multivariable predictive model

The final multivariable model retained six independent preoperative predictors (Table III): ASV reflux (OR, 2.33; 95% CI, 2.03-2.67), incompetent perforators (OR, 2.04; 95% CI, 1.77-2.35), BMI ≥30 (OR, 1.82; 1.60-2.07), deep venous reflux or obstruction (OR, 1.72; 95% CI, 1.44-2.06), CEAP C4-C6 (OR, 1.62; 95% CI, 1.42-1.86), and GSV ≥7 mm (OR, 1.41; 95% CI, 1.23-1.62). Age, sex, diabetes, hypertension, and current smoking did not contribute meaningfully (all |β| < .10 and *P* > .15) and were not retained; their full-model coefficients are reported in Supplementary Table IV (online only). The uniform shrinkage factor was 0.97 (minimal overfitting), no multicollinearity was detected (all variance inflation factors <2.1), and restricted cubic splines for BMI and GSV diameter showed no significant nonlinearity (*P* = .31 and *P* = .26; Supplementary Fig, online only).

**Table III:** Multivariable logistic regression model for 5-year recurrence (N = 10,142)

Predictor	β coefficient	Shrinkage-adjusted β	OR (95% CI)	Shrinkage-adjusted OR	*P* value
Intercept	−2.162	−2.097	–	–	–
BMI ≥30	0.599	0.581	1.82 (1.60-2.07)	1.79	<.001
CEAP C4-C6	0.482	0.468	1.62 (1.42-1.86)	1.60	<.001
ASV reflux^a^	0.846	0.821	2.33 (2.03-2.67)	2.27	<.001
Incompetent perforators	0.713	0.692	2.04 (1.77-2.35)	2.00	<.001
Deep reflux/obstruction	0.542	0.526	1.72 (1.44-2.06)	1.69	<.001
GSV ≥7 mm	0.344	0.334	1.41 (1.23-1.62)	1.40	<.001

*ASV*, Anterior saphenous vein; *BMI*, body mass index; *CEAP*, Clinical, Etiologic, Anatomic, and Pathophysiologic classification; *CI*, confidence interval; *GSV*, great saphenous vein; *OR*, odds ratio.

Uniform shrinkage factor (van Houwelingen-Le Cessie) = 0.97. Primary ORs and 95% CIs are based on unshrunk coefficients with cluster-robust standard errors (patient level). Shrinkage-adjusted ORs = exp(shrinkage-adjusted β); primary inference is based on unshrunk coefficients.

All variance inflation factors <2.1. Restricted cubic splines for BMI and GSV diameter: nonlinearity *P* = .31 and *P* = .26, respectively (Supplementary Fig, online only).

Regression equation (shrinkage adjusted): linear predictor = −2.097 + 0.581 × (BMI ≥30) + 0.468 × (CEAP C4-C6) + 0.821 × (ASV) + 0.692 × (perforators) + 0.526 × (deep) + 0.334 × (GSV ≥7 mm); *P* = 1/(1 + exp [−linear predictor]).

Full multivariable model including all 14 candidate predictors (with nonretained variables: age, sex, diabetes, hypertension, smoking, prior deep vein thrombosis, bilateral disease, and calendar-year) is provided in Supplementary Table IV (online only).

a ASV = anterior saphenous vein (formerly anterior accessory saphenous vein, AASV).

### Model performance

**Fig 2:**
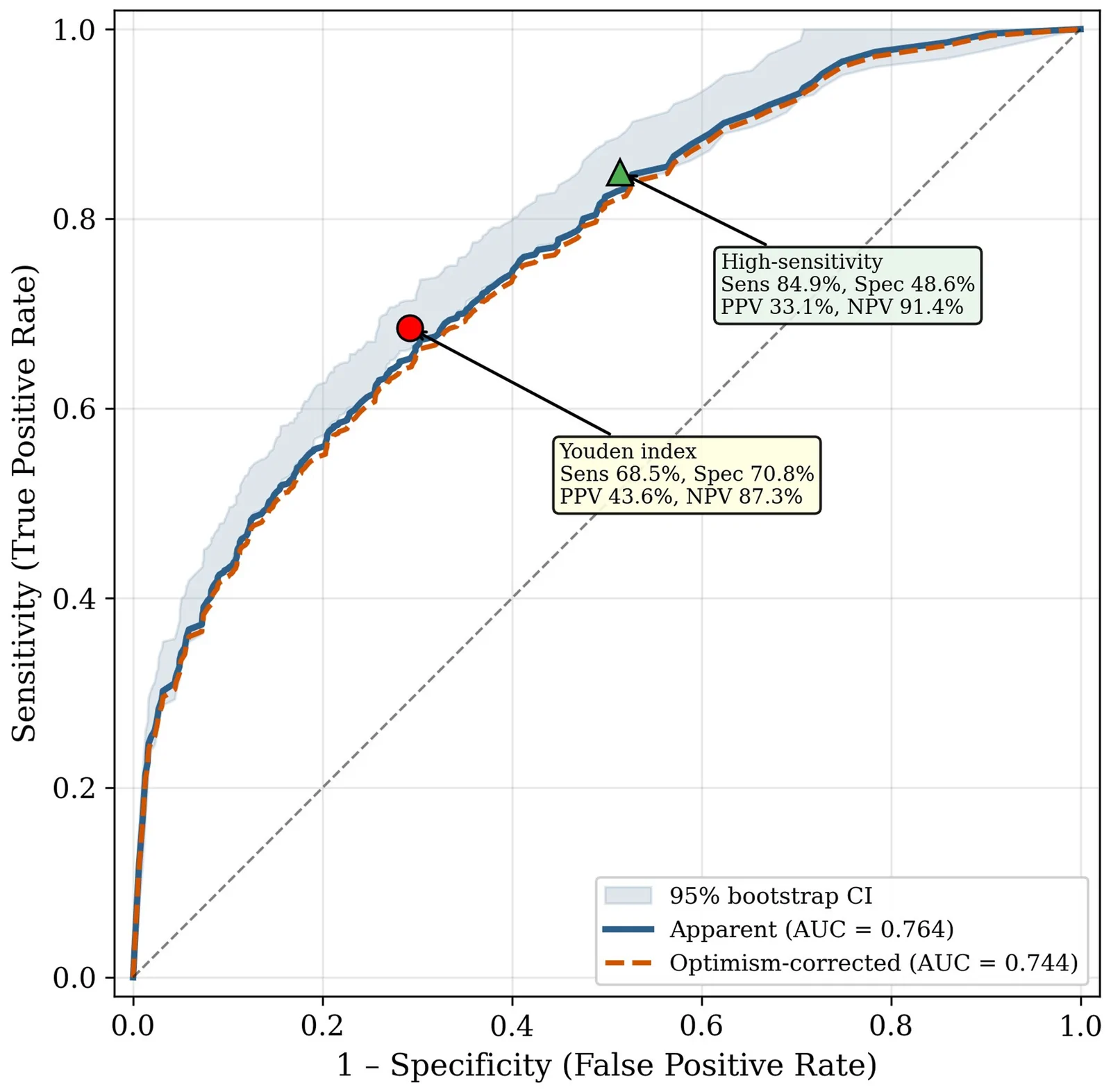
Receiver operating characteristic (ROC) curve. Apparent C-statistic = 0.764 (95% bootstrap confidence interval [*CI*], 0.748-0.780); optimism corrected = 0.744 (500 cluster-bootstrap replications). The Youden-optimal threshold (sensitivity, 68.5%; specificity, 70.8%) corresponds approximately to the moderate/high-risk boundary of the Varicose Vein Recurrence (VVR) Score. Positive predictive value (*PPV*) and negative predictive value (*NPV*) are prevalence dependent and specific to this cohort (baseline recurrence rate, 25.1%); they are reported descriptively only and are not intended for cross-population comparison. *AUC*, area under the curve.

**Fig 3:**
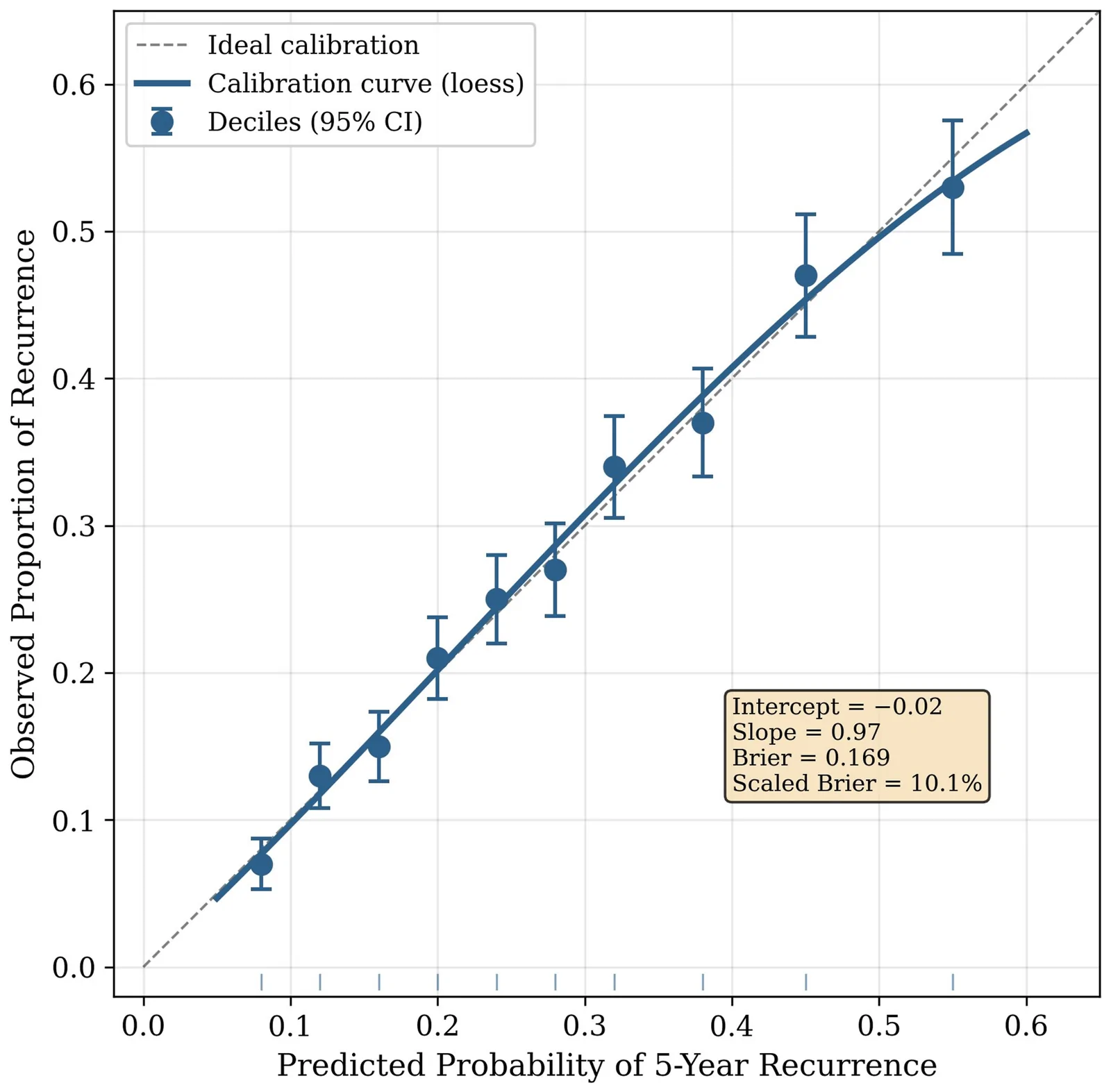
Calibration plot. Points represent deciles of predicted risk with 95% confidence intervals (*CIs*). Calibration intercept = −0.02 (optimism corrected); slope = 0.97 (optimism corrected); apparent Brier score = 0.169; and scaled Brier = 10.1%. The displayed curve represents apparent calibration. The x-axis range (0-0.65) reflects the distribution of predicted probabilities in the cohort.

The apparent C-statistic was 0.764 (95% CI, 0.748-0.780; Fig 2); after cluster bootstrap, the optimism-corrected C-statistic was 0.744 (95% CI, 0.726-0.762; optimism, 0.020). Calibration was excellent (Fig 3): optimism-corrected intercept −0.02 (95% CI, −0.06 to 0.02), slope 0.97 (95% CI, 0.924-1.016). The Brier score was 0.169 (corrected, 0.173; scaled Brier, 10.1%; Brier_null, 0.188). Nagelkerke *R*^2^ was 0.186 (corrected, 0.172). The continuous model improved discrimination only marginally (C-statistic difference, 0.008; *P* = .18), confirming minimal information loss from dichotomization. At the Youden-optimal threshold (predicted probability, 0.25), sensitivity was 68.5% and specificity was 70.8%; positive predictive value (43.6%) and negative predictive value (87.3%) are specific to the 25.1% baseline prevalence. Decision-curve analysis indicated net benefit exceeding both treat-all and treat-none strategies across threshold probabilities of approximately 10% to 35%; detailed visualization and plain-language interpretation are provided in the Discussion section.

### Clinical risk scoring system (VVR score)

**Fig 4:**
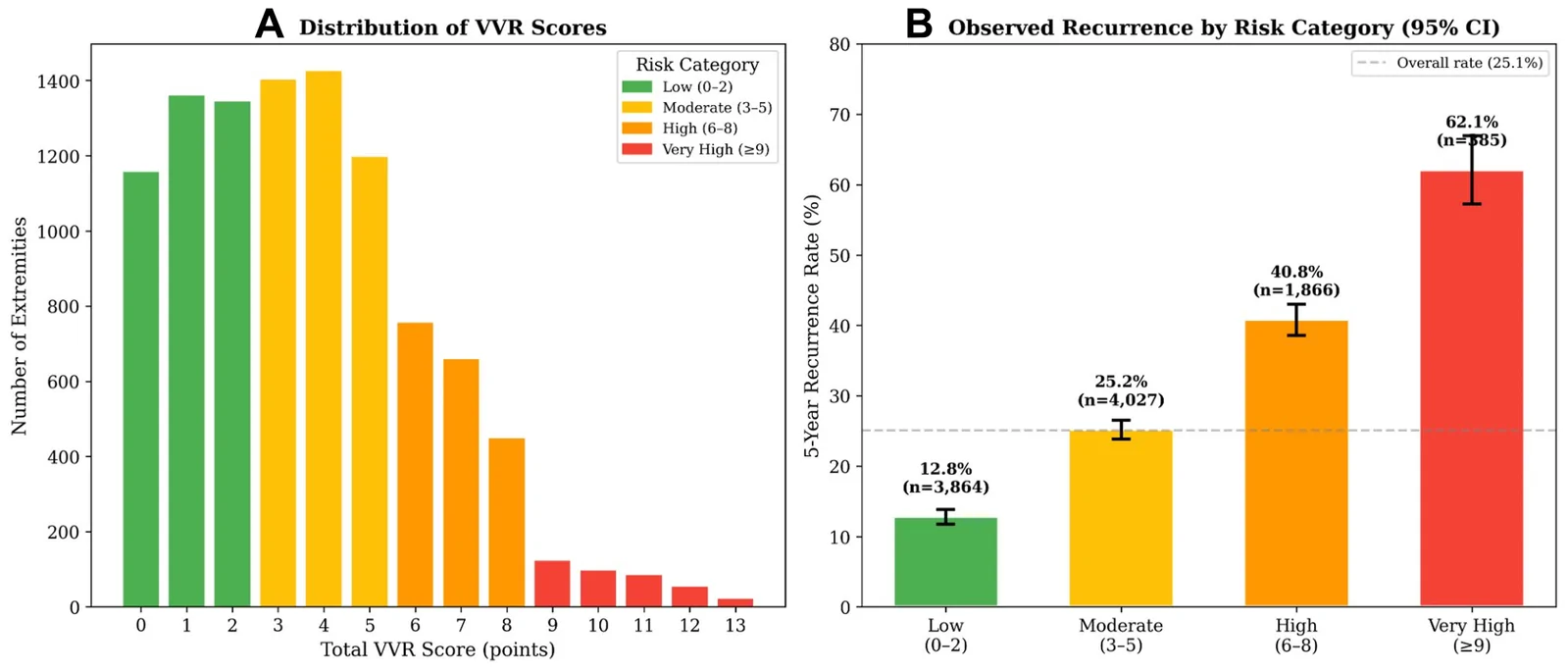
**A,** Distribution of Varicose Vein Recurrence (*VVR*) Scores, color coded by risk category. **B,** Observed 5-year recurrence rates with 95% confidence intervals (*CIs*) by risk category. The dashed line indicates the overall recurrence rate (25.1%).

Shrinkage-adjusted coefficients were converted into an integer-based scoring system (Table IV): BMI ≥30 (2 points), CEAP C4-C6 (2 points), ASV reflux (3), incompetent perforators (3), deep venous reflux/obstruction (2), GSV ≥7 mm (1); total 0 to 13. Four risk categories were defined (Table V): low risk (0-2 points; n = 3864, 495 recurrences, 12.8%; 95% CI, 11.8-13.9), moderate (3-5; n = 4027, 1015 recurrences, 25.2%; 95% CI, 23.9-26.5), high (6-8; n = 1866, 779 recurrences, 41.7%; 95% CI, 39.5-43.9), and very high (≥9; n = 385, 257 recurrences, 66.8%; 95% CI, 61.9-71.3). Counts sum to 2546 recurrences across 10,142 extremities, matching the primary-cohort total. Within-category absolute observed-predicted differences ranged from +0.7 to +1.8 percentage points (Fig 4); cutoffs were prespecified for monotonic risk increase, adequate group size (≥4% of cohort), and alignment with predicted-probability thresholds (∼15%, ∼25%, and ∼40%). For external validation and precise individual risk estimation, the full regression equation (Table III; Supplementary Table IV, online only) should be used. Detailed performance metrics for both the dichotomized and continuous-predictor models are presented in Table VI.

**Table IV:** Varicose Vein Recurrence (VVR) Scoring system

Predictor	Definition	Points	Data source
BMI ≥30	BMI ≥30 kg/m^2^	2	Preoperative measurement
CEAP C4-C6	Skin changes, lipodermatosclerosis, or healed/active ulceration	2	Clinical classification
ASV reflux^a^	Anterior saphenous vein (formerly anterior accessory saphenous vein, AASV) reflux on duplex	3	Preoperative duplex US
Incompetent perforators	Pathological perforating veins (≥3.5 mm, reflux >0.5 seconds)	3	Preoperative duplex US
Deep venous reflux/obstruction	Deep system reflux or post-thrombotic changes on duplex	2	Preoperative duplex US
GSV ≥7 mm	GSV diameter ≥7 mm at 3-5 cm distal to SFJ	1	Preoperative duplex US
Total score range		0-13	

*ASV*, Anterior saphenous vein; *BMI*, body mass index; *CEAP*, Clinical, Etiologic, Anatomic, and Pathophysiologic classification; *GSV*, great saphenous vein; *SFJ*, saphenofemoral junction; *US*, ultrasound.

Point assignment: The smallest shrinkage-adjusted coefficient (GSV ≥7 mm; β = .334) was assigned one point (reference unit). Other coefficients were scaled proportionally and rounded to the nearest integer: BMI 1.74→2, CEAP 1.40→2, ASV 2.46→3, perforators 2.07→3, and deep 1.57→2.

Score-to-probability mapping is approximate because of integer rounding and aggregation of heterogeneous predictor combinations onto a single score. Exact individual risk should be computed using the regression equation in Table III; Supplementary Table IV (online only).

Approximate score-to-probability conversion using the average shrinkage-adjusted β per point (Σβ/Σpoints = 3.422/13 ≈ 0.263), with intercept −2.097: *P* (score) = 1/(1 + exp [2.097 − 0.263 × score]).

Score → predicted 5-year recurrence probability: 0 → 10.9%; 1 → 13.8%; 2 → 17.2%; 3 → 21.3%; 4 → 26.0%; 5 → 31.4%; 6 → 37.3%; 7 → 43.7%; 8 → 50.2%; 9 → 56.8%; 10 → 63.1%; 11 → 69.0%; and 12 → 74.3%; 13 → 79.0%. Empirically observed recurrence rates within risk categories (Table V) are 12.8%, 25.2%, 41.7%, and 66.8%, broadly consistent with the equation-derived mapping at category-midpoint scores (1, 4, 7, and 11). The equation slightly overpredicts at higher scores by 2.0 to 2.2 percentage points; this minor divergence reflects the integer-rounded scoring system aggregating heterogeneous predictor combinations onto a single point total. The underlying logistic model is well calibrated overall (calibration intercept, −0.02; slope, 0.97; Table VI).

a ASV = anterior saphenous vein (formerly anterior accessory saphenous vein [AASV]); terminology updated to align with current consensus nomenclature.

**Table V:** Risk stratification: Observed vs predicted recurrence (N = 10,142)

Risk category	Score	N (%)	Observed recurrences (rate %, 95% CI)	Mean predicted risk (%)	O-P difference (pp)
Low	0-2	3864 (38.1)	495/12.8 (11.8-13.9)	11.9	+0.9
Moderate	3-5	4027 (39.7)	1015/25.2 (23.9-26.5)	24.0	+1.2
High	6-8	1866 (18.4)	779/41.7 (39.5-43.9)	41.0	+0.7
Very high	≥9	385 (3.8)	257/66.8 (61.9-71.3)	65.0	+1.8
Total	0-13	10,142 (100.0)	2546/25.1 (24.3-25.9)	25.1	0.0

*CI*, Confidence interval.

95% CIs for observed recurrence rates calculated using the Wilson method. O-P = observed minus predicted percentage-point difference. Counts are low 495/3864; moderate 1015/4027; high 779/1866; very high 257/385; and total 2546/10,142 (sum verified internally consistent with primary-cohort recurrence count).

Risk-category cutoffs were selected a priori to provide clinically interpretable strata with monotonic risk increase and adequate group sizes (minimum, ∼4% of the cohort).

Hypothetical follow-up scenarios by risk category are presented in the Supplementary Materials (online only) for illustrative purposes only; they are not clinical recommendations and require prospective validation and cost-effectiveness evaluation.

**Table VI:** Model performance: Dichotomized vs continuous-predictor models (N = 10,142)

Performance metric	Dichotomized apparent (95% CI)	Dichotomized optimism-corrected (95% CI)	Continuous apparent (95% CI)	Continuous optimism-corrected (95% CI)
Discrimination				
C-statistic	0.764 (0.748-0.780)	0.744 (0.726-0.762)	0.772 (0.756-0.788)	0.752 (0.734-0.770)
Discrimination slope	0.146	0.137	0.160	0.151
Somers' Dxy	0.528	0.488	0.544	0.504
Calibration				
Calibration intercept (α)	0.000	−0.02 (−0.06 to 0.02)	0.000	−0.01 (−0.05 to 0.03)
Calibration slope (β)	1.000	0.970 (0.924-1.016)	1.000	0.975 (0.931-1.019)
Overall				
Brier score	0.169	0.173	0.167	0.171
Scaled Brier score	10.1%	8.0%	11.2%	9.0%
Nagelkerke *R*^2^	0.186	0.172	0.200	0.185
Clinical utility				
Net benefit at 20% threshold	0.070	0.065	0.073	0.068

*CI*, Confidence interval.

Internal validation: cluster bootstrap at the patient level (500 replications). 95% CIs for optimism-corrected metrics derived from the bootstrap distribution (percentile method).

Scaled Brier = (Brier_null − Brier_model)/Brier_null, where Brier_null = prevalence × (1 − prevalence) = 0.251 × 0.749 = 0.188.

The continuous model retains body mass index (BMI) and great saphenous vein (GSV) diameter as continuous variables; the dichotomized model uses BMI ≥30 and GSV ≥7 mm.

C-statistic difference (continuous − dichotomized): 0.008 (95% CI, −0.004 to 0.020; *P* = .18).

### Sensitivity analyses

Nine prespecified sensitivity analyses (Supplementary Table VII, online only) produced consistent results with C-statistics 0.71 to 0.75: (1) IPCW on all 12,480 extremities (C = 0.75, near-identical ORs); (2) Cox regression (Harrell C, 0.73; corrected, 0.71; concordant hazard ratios); (3) recurrences ≤12 months excluded (C = 0.75); (4) reintervention as end point (C = 0.73, supporting robustness to verification bias); (5) generalized estimating equation (concordant, standard error within 3%); (6) era-adjusted model (no significant era × predictor interactions, all *P* > .15); (7) MICE imputation (coefficients within 2%); (8) treatment-type covariate (Wald *P* = .18; ΔC = +0.003; coefficient changes ≤4%); and (9) continuous calendar-year analysis with time × predictor interactions (smallest *P* = .21). Procedure-stratified subgroup analysis (Supplementary Table V, online only) showed preserved predictor direction and magnitude across operative subgroups, with apparent C-statistics of 0.74 (HL + stripping, n = 7099), 0.72 (HL alone, n = 1521), 0.73 (overlapping SPJ-procedure subgroup, n = 811), and 0.70 (phlebectomy alone, n = 1522), and calibration slopes 0.94 to 0.98 with intercepts −0.05 to +0.04 (subgroup sample sizes precluded reliable within-stratum bootstrap optimism correction). The 5-year recurrence rate was 26.6% (404/1521) in HL alone vs 24.7% (1755/7099) in HL + stripping, consistent with selective use of HL alone in patients with limited above-knee truncal reflux (Table I).

## Discussion

In this 20-year single-center cohort of over 10,000 operated extremities, we developed and internally validated the VVR Score, which combines six routinely available preoperative variables to estimate 5-year recurrence risk after open superficial venous surgery. Internal validation demonstrated moderate discrimination (optimism-corrected C-statistic, 0.744) with excellent calibration (slope, 0.97; intercept, −0.02), and the score stratified patients into four clinically distinct categories with observed 5-year recurrence rates spanning 12.8% to 66.8%. Model estimates reflect recurrence rates under our follow-up intensity (structured duplex at 6 and 12 months, then symptom-triggered or annual-clinical-suspicion-driven thereafter); external cohorts with different surveillance protocols may require recalibration of absolute risk estimates.

ASV reflux and incompetent perforators emerged as the strongest independent predictors. The ASV is an underappreciated reflux pathway that can sustain or re-establish reflux even after adequate GSV treatment^12^^,^^42^^,^^43^; the OR of 2.33 underscores the importance of systematic preoperative duplex assessment, including separate ostial identification.^44^^,^^45^ BMI ≥30 likely reflects higher venous pressures and more advanced underlying disease^46^^,^^47^; advanced CEAP class identifies extensive disease that is inherently more recurrence prone; deep venous reflux/obstruction sustains ambulatory venous hypertension that drives superficial disease.^48^^,^^49^ Mechanism-specific analyses (Supplementary Table VIII, online only) showed ASV reflux and perforators particularly predictive of neovascularization-type and perforator-mediated recurrence, whereas BMI and CEAP were more uniformly associated across mechanisms.

The moderate discrimination (C-statistic, 0.744) is expected because key determinants of recurrence—neovascularization, intraoperative technical factors, and postoperative compression adherence—are not measurable preoperatively and cannot be captured by any preoperative model. This level is typical for preoperative prediction models based on routinely available variables^35^; higher discrimination would require intraoperative or postoperative data, defeating the purpose of a counseling tool. Calibration is therefore the primary metric: the near-zero intercept and slope close to unity suggest that absolute risk estimates are reliable in our system, although recalibration may be needed in external cohorts.^50^ Positive predictive value and negative predictive value are prevalence dependent and reported only as descriptive performance summaries at one threshold; they are not intended for cross-population comparison.

### Interpreting the decision curve analysis

**Fig 5:**
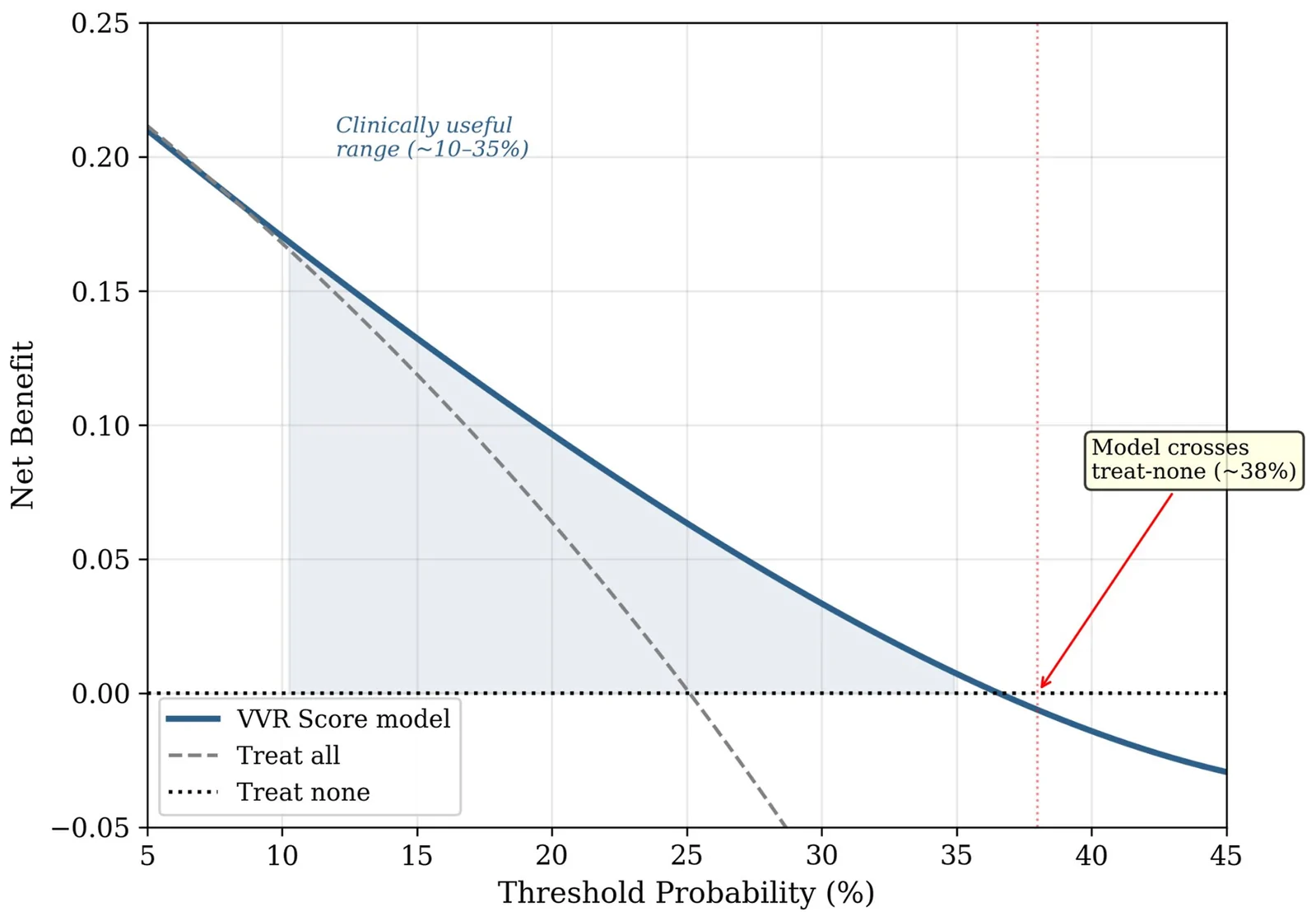
Decision curve analysis. The Varicose Vein Recurrence (*VVR*) Score model shows net benefit exceeding the treat-all and treat-none strategies across threshold probabilities of approximately 10% to 35%. In this clinical context, “treat-all” refers to assigning intensified surveillance to every operated patient regardless of risk score, and “treat-none” refers to relying entirely on symptom-driven representation. The annotated crossing point (∼38%) indicates the threshold above which the model no longer provides net benefit over treating no patients. The x-axis range (5%-45%) reflects clinically plausible thresholds; optimal threshold selection depends on local prevalence and clinical trade-off preferences. A plain-language interpretation is provided in the Discussion section.

Decision curve analysis (Fig 5) shows that across the clinically relevant 10% to 35% threshold range—the band of preoperative risks at which a clinician might consider intensified surveillance worthwhile—the VVR Score yields net benefit superior to “treat-all” and “treat-none” reference strategies. In plain terms, the score adds clinical value primarily for moderate- and high-risk patients, where surveillance can be intensified, and supports reduced-intensity follow-up for clearly low-risk patients without unduly increasing missed recurrences.

### Procedure-type considerations

The score performed comparably across the three mutually exclusive operative subgroups (Supplementary Table V, online only) with apparent C-statistics of 0.70 to 0.74 (subgroup sample sizes precluded reliable within-stratum bootstrap optimism correction). The HL-alone subgroup recurrence rate was 26.6% (404/1521), modestly higher than HL + stripping (24.7%; 1755/7099), consistent with selective use of HL alone in patients with limited above-knee truncal reflux. Although HL alone is no longer recommended in current guidelines,^25^ we retained this subgroup for transparency about the case-mix of our 20-year cohort; preserved discrimination supports score use in the contemporary HL + stripping setting and, with appropriate caution, in SPJ-procedure or phlebectomy-only settings.^36^^,^^51^

### Comparison with prior work

Compared with the recent Hu et al^20^ nomogram (n = 856, no reported optimism-corrected internal validation), the VVR Score offers a substantially larger derivation cohort, prespecified Transparent Reporting of a Multivariable Prediction Model for Individual Prognosis or Diagnosis-compliant analysis, and an integer-based bedside instrument that does not require an electronic calculator while remaining anchored to a fully transparent regression equation (Supplementary Table IV, online only) for precise risk estimation in research settings.

### Limitations

Limitations include (1) retrospective single-center design (era-stratified and continuous-time analyses showed no significant calendar × predictor interaction); (2) contemporary applicability—HL alone (15% of cohort) is no longer guideline recommended,^25^ and applicability to endothermal, nonthermal/nontumescent, and foam-sclerotherapy cohorts is unknown and requires rederivation or recalibration; (3) loss to follow-up before 5 years (18.7%), partly addressed by IPCW and Cox sensitivity analyses; (4) the management-dependent component of the composite outcome introduces verification bias not fully resolved despite the consistent reintervention-end point sensitivity analysis (C-statistic, 0.73); (5) the reintervention-modality mix shifted across eras (era 1 open 82.6% → era 3 41.3%) as endovenous techniques became locally available, with preserved discrimination across eras supporting model robustness; (6) dichotomization of BMI and GSV diameter entails marginal information loss (continuous-predictor C-statistic improvement, 0.008; *P* = .18). The PROBAST-guided assessment (Supplementary Table IX, online only) identifies outcome ascertainment, single-center design, and contemporary applicability as the principal residual issues.

### Future directions

External validation in geographically diverse open-surgical cohorts is the highest priority. Separate validation—with prespecified recalibration where calibration deviates from ideal—should be undertaken in endovenous and foam-sclerotherapy cohorts, since dominant recurrence mechanisms differ across modalities. Pragmatic implementation studies could test whether score-guided surveillance reduces missed recurrences and resource utilization. If external performance is preserved, a web- or app-based calculator using the full regression equation can be developed.

## Conclusions

The VVR Score provides an internally validated tool for estimating 5-year recurrence risk using six preoperative clinical and duplex variables (BMI, CEAP class, ASV reflux, incompetent perforators, deep venous reflux or obstruction, and GSV diameter) after open superficial venous surgery. Performance is preserved across operative subgroups and for the harder reintervention end point. External validation in open-surgical cohorts and rederivation/recalibration before use in endovenous or foam-sclerotherapy populations are essential next steps. Pending these, the VVR Score is suitable for structured preoperative counseling, research stratification, and risk-stratified follow-up planning within open-surgical practice.

## Author Contributions

Conception and design: DN, KP, VM, SB, NB, MN

Analysis and interpretation: DN

Data collection: DN, KP, VM, SB, NB, MN

Writing the article: DN

Critical revision of the article: DN, KP, VM, SB, NB, MN

Final approval of the article: DN, KP, VM, SB, NB, MN

Statistical analysis: DN

Obtained funding: DN

Overall responsibility: DN

## Funding

None.

## Data availability

The anonymized data set is available from the corresponding author upon reasonable request, subject to institutional data governance policies.

## Disclosures

None.

## Figures and Tables

**Supplementary Fig:**
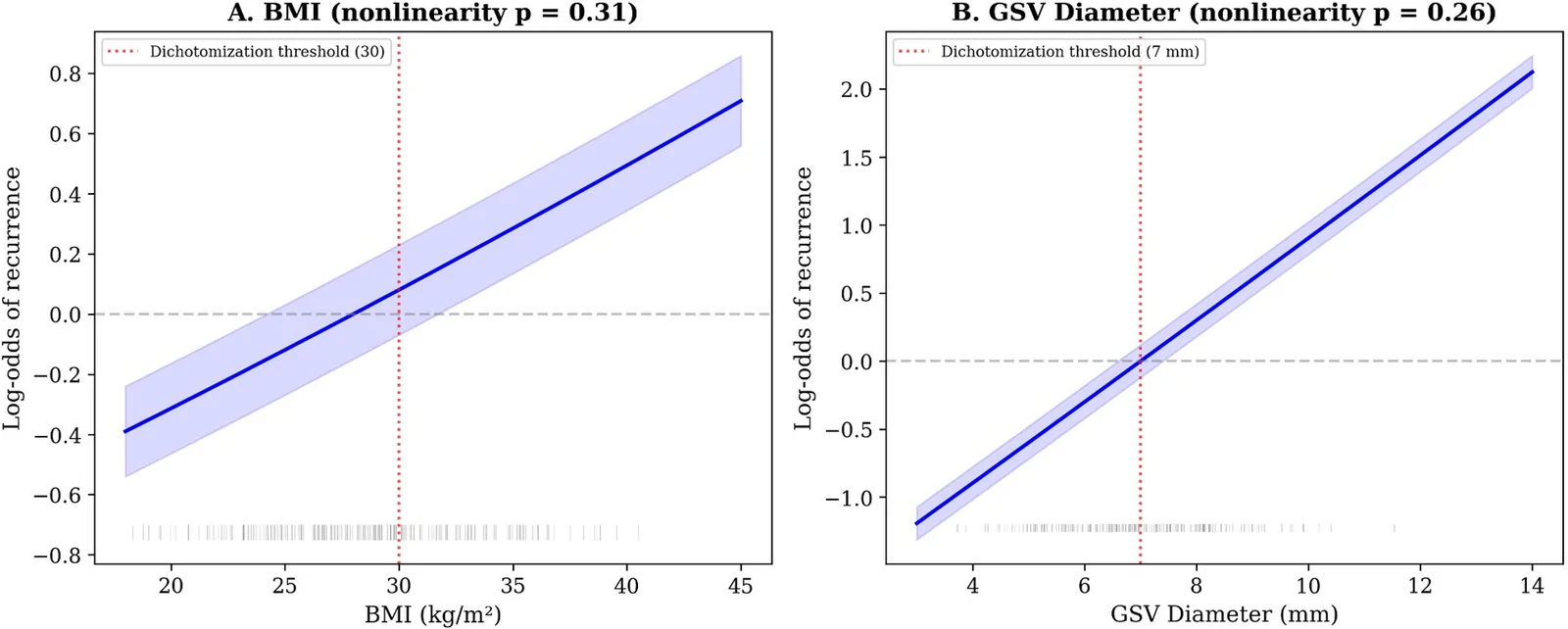


## References

[1] Rabe E., Guex J.J., Puskas A., Scuderi A., Fernandez Quesada F., VCP Coordinators (2012). Epidemiology of chronic venous disorders in geographically diverse populations: results from the Vein Consult Program. Int Angiol.

[2] Beebe-Dimmer J.L., Pfeifer J.R., Engle J.S., Schottenfeld D. (2005). The epidemiology of chronic venous insufficiency and varicose veins. Ann Epidemiol.

[3] Gloviczki P., Comerota A.J., Dalsing M.C. (2011). The care of patients with varicose veins and associated chronic venous diseases: clinical practice guidelines of the Society for Vascular Surgery and the American Venous Forum. J Vasc Surg.

[4] Wittens C., Davies A.H., Bækgaard N. (2015). Editor’s choice—management of chronic Venous Disease: clinical practice guidelines of the European Society for Vascular Surgery (ESVS). Eur J Vasc Endovasc Surg.

[5] Fischer R., Linde N., Duff C., Jeanneret C., Chandler J.G., Seeber P. (2001). Late recurrent saphenofemoral junction reflux after ligation and stripping of the greater saphenous vein. J Vasc Surg.

[6] Perrin M.R., Guex J.J., Ruckley C.V., REVAS group (2000). Recurrent varices after surgery (REVAS), a consensus document. Cardiovasc Surg.

[7] van Rij A.M., Jiang P., Solomon C., Christie R.A., Hill G.B. (2003). Recurrence after varicose vein surgery: a prospective long-term clinical study. J Vasc Surg.

[8] Winterborn R.J., Foy C., Earnshaw J.J. (2004). Causes of varicose vein recurrence: late results of a randomized controlled trial of stripping the long saphenous vein. J Vasc Surg.

[9] De Maeseneer M.G., Tielliu I.F., Van Schil P.E., De Hert S.G., Eyskens E.J. (1999). Clinical relevance of neovascularisation on duplex ultrasound in the long-term follow-up after varicose vein operation. Phlebology.

[10] Stucker M., Netz K., Breuckmann F., Altmeyer P., Mumme A. (2004). Histomorphologic classification of recurrent saphenofemoral reflux. J Vasc Surg.

[11] Egan B., Donnelly M., Bresnihan M., Tierney S., Feeley M. (2006). Neovascularization: an "innocent bystander" in recurrent varicose veins. J Vasc Surg.

[12] Fischer R., Linde N., Duff C. (2005). Neovascularization after great saphenous vein surgery. Phlebology.

[13] Labropoulos N., Giannoukas A.D., Delis K. (1997). Where does venous reflux start?. J Vasc Surg.

[14] Perrin M.R., Labropoulos N., Leon L.R. (2006). Presentation of the patient with recurrent varices after surgery (REVAS). J Vasc Surg.

[15] Rasmussen L.H., Bjoern L., Lawaetz M., Blemings A., Lawaetz B., Eklof B. (2010). Randomised clinical trial comparing endovenous laser ablation with stripping of the great saphenous vein: clinical outcome and recurrence after 2 years. Eur J Vasc Endovasc Surg.

[16] O’Donnell T.F., Balk E.M., Dermody M., Tangney E., Iafrati M.D. (2016). Recurrence of varicose veins after endovenous ablation of the great saphenous vein in randomized trials. J Vasc Surg Venous Lymphat Disord.

[17] Kostas T., Ioannou C.V., Touloupakis E. (2004). Recurrent varicose veins after surgery: a new appraisal of a common and complex problem in vascular surgery. Eur J Vasc Endovasc Surg.

[18] Stuart W.P., Adam D.J., Allan P.L., Ruckley C.V., Bradbury A.W. (2000). The relationship between the number, competence, and diameter of medial calf perforating veins and clinical status. J Vasc Surg.

[19] Farah M.H., Nayfeh T., Urtecho M. (2022). A systematic review supporting the Society for Vascular Surgery, the American Venous Forum, and the American Vein and Lymphatic Society guidelines on the management of varicose veins. J Vasc Surg Venous Lymphat Disord.

[20] Hu H., Hu L., Deng Z., Jiang Q. (2024). A prognostic nomogram for recurrence survival in post-surgical patients with varicose veins of the lower extremities. Sci Rep.

[21] De Maeseneer M.G. (2024). The role of postoperative neovascularisation in recurrence of varicose veins: two decades of follow-up. Phlebology.

[22] Collins G.S., Reitsma J.B., Altman D.G., Moons K.G. (2015). Transparent reporting of a multivariable prediction model for individual prognosis or diagnosis (TRIPOD): the TRIPOD statement. BMJ.

[23] Moons K.G.M., Altman D.G., Reitsma J.B. (2015). Transparent Reporting of a multivariable prediction model for Individual Prognosis Or Diagnosis (TRIPOD): explanation and elaboration. Ann Intern Med.

[24] World Medical Association (2013). WMA declaration of Helsinki–ethical principles for medical research involving human subjects (2013 amendment). JAMA.

[25] De Maeseneer M.G., Kakkos S.K., Aherne T., ESVS Guidelines Committee (2022). Editor’s choice—European Society for Vascular Surgery (ESVS) 2022 clinical practice guidelines on the management of chronic venous disease of the lower limbs. Eur J Vasc Endovasc Surg.

[26] Coleridge-Smith P., Labropoulos N., Partsch H., Myers K., Nicolaides A., Cavezzi A. (2006). Duplex ultrasound investigation of the veins in chronic venous disease of the lower limbs–UIP consensus document. Part I. Eur J Vasc Endovasc Surg.

[27] Labropoulos N., Tiongson J., Pryor L. (2003). Definition of venous reflux in lower-extremity veins. J Vasc Surg.

[28] Eklöf B., Rutherford R.B., Bergan J.J., American Venous Forum International Ad Hoc Committee for Revision of the CEAP Classification (2004). Revision of the CEAP classification for chronic venous disorders: consensus statement. J Vasc Surg.

[29] Sandri J.L., Barros F.S., Pontes S., Jacques C., Salles-Cunha S.X. (1999). Diameter-reflux relationship in perforating veins of patients with varicose veins. J Vasc Surg.

[30] van Buuren S., Groothuis-Oudshoorn K. (2011). Mice: multivariate imputation by chained equations in R. J Stat Softw.

[31] Peduzzi P., Concato J., Kemper E., Holford T.R., Feinstein A.R. (1996). A simulation study of the number of events per variable in logistic regression analysis. J Clin Epidemiol.

[32] Riley R.D., Ensor J., Snell K.I.E. (2020). Calculating the sample size required for developing a clinical prediction model. BMJ.

[33] Liang K.Y., Zeger S.L. (1986). Longitudinal data analysis using generalized linear models. Biometrika.

[34] Steyerberg E.W., Vergouwe Y. (2014). Towards better clinical prediction models: seven steps for development and an ABCD for validation. Eur Heart J.

[35] Steyerberg E.W. (2019). Clinical prediction models: a practical approach to development, validation, and updating.

[36] Steyerberg E.W., Vickers A.J., Cook N.R. (2010). Assessing the performance of prediction models: a framework for some traditional and novel measures. Epidemiology.

[37] Harrell F.E. (2015). Regression modeling strategies.

[38] Vickers A.J., Elkin E.B. (2006). Decision curve analysis: a novel method for evaluating prediction models. Med Decis Making.

[39] Van Calster B., Wynants L., Verbeek J.F.M. (2018). Reporting and interpreting decision curve analysis: a guide for investigators. Eur Urol.

[40] Steyerberg E.W., Harrell F.E., Borsboom G.J., Eijkemans M.J., Vergouwe Y., Habbema J.D. (2001). Internal validation of predictive models: efficiency of some procedures for logistic regression analysis. J Clin Epidemiol.

[41] Van Houwelingen J.C., Le Cessie S. (1990). Predictive value of statistical models. Stat Med.

[42] De Maeseneer M.G. (2004). The role of postoperative neovascularisation in recurrence of varicose veins. Acta Chir Belg.

[43] Pichot O., Kabnick L.S., Creton D., Merchant R.F., Schuller-Petrovic S., Chandler J.G. (2004). Duplex ultrasound scan findings two years after great saphenous vein radiofrequency obliteration. J Vasc Surg.

[44] De Maeseneer M.G., Philipsen T.E., Vandenbroeck C.P. (2007). Closure of the cribriform fascia: an efficient anatomical barrier against postoperative neovascularisation at the saphenofemoral junction? A prospective study. Eur J Vasc Endovasc Surg.

[45] Labropoulos N., Mansour M.A., Kang S.S., Gloviczki P., Baker W.H. (1999). New insights into perforator vein incompetence. Eur J Vasc Endovasc Surg.

[46] Willenberg T., Schumacher A., Amann-Vesti B. (2010). Impact of obesity on venous hemodynamics of the lower limbs. J Vasc Surg.

[47] Davies H.O., Popplewell M., Singhal R., Smith N., Bradbury A.W. (2017). Obesity and lower limb venous disease–the epidemic of phlebesity. Phlebology.

[48] Labropoulos N., Volteas N., Leon M. (1997). The role of venous outflow obstruction in patients with chronic venous dysfunction. Arch Surg.

[49] Johnson B.F., Manzo R.A., Bergelin R.O., Strandness D.E. (1995). Relationship between changes in the deep venous system and the development of the post-thrombotic syndrome after an acute episode of lower limb deep vein thrombosis. J Vasc Surg.

[50] Van Calster B., McLernon D.J., van Smeden M., Wynants L., Steyerberg E.W. (2019). Calibration: the Achilles heel of predictive analytics. BMC Med.

[51] Brake M., Lim C.S., Shepherd A.C., Shalhoub J., Davies A.H. (2013). Pathogenesis and etiology of recurrent varicose veins. J Vasc Surg.

