# Supplementary material for: Development and internal validation of a new Varicose Vein Recurrence Score for open superficial venous surgery
Source: J Vasc Surg Venous Lymphat Disord. 2026 Jul 24;14(6):102583. doi: 10.1016/j.jvsv.2026.102583 (PMC13495597; doi:10.1016/j.jvsv.2026.102583)
Supplement: Supplementary file 1 [file mmc1.docx]

**Supplementary Materials**

*Development and Internal Validation of a New Varicose Vein Recurrence Score for Open Superficial Venous Surgery (VVR Score)*

*Revision R1 — May 2026*

## **Supplementary Table S1. Operative Characteristics by Calendar Era (N = 10,142)**

| **Characteristic** | **Era 1 (2006–2012, n=4,218)** | **Era 2 (2013–2018, n=3,612)** | **Era 3 (2019–2025, n=2,312)** |
| --- | --- | --- | --- |
| HL + stripping, n (%) | 2,985 (70.8) | 2,514 (69.6) | 1,600 (69.2) |
| HL alone, n (%) | 633 (15.0) | 541 (15.0) | 347 (15.0) |
| SPJ procedures, n (%) | 337 (8.0) | 289 (8.0) | 185 (8.0) |
| SPJ stripping, n (%)* | 102 (30.3) | 94 (32.5) | 60 (32.4) |
| SPJ ligation only, n (%)* | 235 (69.7) | 195 (67.5) | 125 (67.6) |
| Phlebectomy alone, n (%) | 600 (14.2) | 557 (15.4) | 365 (15.8) |
| Cribriform fascia closure, n (%) | 421 (10.0) | 3,251 (90.0) | 2,081 (90.0) |
| Stripping to knee, n (%)† | 2,836 (96.0) | 2,388 (95.5) | 1,520 (95.8) |
| Concomitant perforator Tx, n (%) | 928 (22.0) | 1,047 (29.0) | 786 (34.0) |
| Open subfascial ligation, n (%)‡ | 742 (80.0) | 486 (46.4) | 141 (17.9) |
| Open epifascial ligation, n (%)‡ | 186 (20.0) | 283 (27.0) | 102 (13.0) |
| Foam sclerotherapy of perforator, n (%)‡ | 0 (0) | 189 (18.1) | 321 (40.8) |
| Endothermal perforator ablation, n (%)‡ | 0 (0) | 89 (8.5) | 222 (28.2) |
| 5-year recurrence rate (%) | 26.8 | 24.5 | 23.1 |
| 5-year reintervention rate (%) | 19.8 | 18.1 | 17.2§ |
| Open reintervention, % of all reint. | 82.6 | 54.7 | 41.3 |
| Endothermal reintervention, % | 6.2 | 24.5 | 38.1 |
| Foam sclerotherapy, % | 11.2 | 20.8 | 20.6 |

** Among SPJ procedures only. † Among HL + stripping procedures only. ‡ Among extremities receiving any perforator treatment within each era. § Era 3 estimates may be less stable due to shorter maximum follow-up (see main text).*

Cribriform fascia closure was adopted as standard practice from 2013 onward following neovascularisation literature emerging in the late 2000s. Concomitant perforator treatment increased progressively across eras and shifted in technique from open subfascial ligation toward minimally invasive endothermal and foam approaches. Reintervention modality similarly shifted from predominantly open in Era 1 (82.6%) to predominantly endovenous in Era 3 (38.1% endothermal, 20.6% foam, 41.3% open). Despite these substantial operative and reintervention-modality changes, predictor effects of the six retained variables did not differ significantly between eras (era × predictor interactions all p > 0.15; Table S9).

## **Supplementary Table S2. Missing Data by Predictor Variable (N = 12,480)**

| **Variable** | **Missing, n (%)** | **Pattern** | **Imputation strategy** |
| --- | --- | --- | --- |
| BMI | 187 (1.5) | MCAR | MICE (m=20) |
| CEAP class | 0 (0) | — | Required at intake |
| ASV reflux on duplex | 423 (3.4) | MAR (era-related) | MICE (m=20) |
| Perforator status | 312 (2.5) | MAR (era-related) | MICE (m=20) |
| Deep system status | 0 (0) | — | Required at intake |
| GSV diameter | 156 (1.3) | MCAR | MICE (m=20) |
| Outcome (5-yr recurrence) | 2,338 (18.7) | MAR (era, distance) | IPCW + Cox sensitivity |

MAR = missing at random; MCAR = missing completely at random. All six core predictors were complete in 11,843 extremities (94.9%). MICE (m = 20) yielded coefficients within 2% of the complete-case analysis, supporting the primary complete-case approach.

## **Supplementary Table S3. Baseline Characteristics: Retained vs. Lost to Follow-up**

| **Characteristic** | **Retained (n=10,142)** | **Lost to f/u (n=2,338)** | **SMD** |
| --- | --- | --- | --- |
| Age, mean (SD), years | 52.4 (12.1) | 52.9 (12.6) | 0.04 |
| Female sex, n (%) | 6,289 (62.0) | 1,425 (61.0) | 0.02 |
| BMI ≥30, n (%) | 3,144 (31.0) | 699 (29.9) | 0.02 |
| CEAP C4–C6, n (%) | 2,738 (27.0) | 631 (27.0) | <0.01 |
| ASV reflux, n (%) | 2,332 (23.0) | 524 (22.4) | 0.01 |
| Incompetent perforators, n (%) | 3,549 (35.0) | 795 (34.0) | 0.02 |
| Deep reflux/obstruction, n (%) | 811 (8.0) | 194 (8.3) | 0.01 |
| GSV ≥7 mm, n (%) | 4,564 (45.0) | 1,028 (44.0) | 0.02 |
| Surgical era 1 (2006–2012), n (%) | 4,218 (41.6) | 976 (41.7) | <0.01 |

SMD = standardized mean difference. All SMDs <0.10, indicating no systematic baseline differences between groups retained in the primary analysis (n = 10,142) and those lost to follow-up before 5 years (n = 2,338).

## **Supplementary Table S4. Full Multivariable Model Including Non-Retained Candidate Predictors**

Linear predictor (LP) of the final shrinkage-adjusted six-variable model:

*LP = −2.097 + 0.581 × (BMI ≥30) + 0.468 × (CEAP C4–C6) + 0.821 × (ASV reflux) + 0.692 × (incompetent perforators) + 0.526 × (deep reflux/obstruction) + 0.334 × (GSV ≥7 mm).*

Predicted 5-year recurrence probability: p = 1 / (1 + exp(−LP)). Use the exact equation for individual risk estimates; the integer-based VVR Score (Table 4) provides a clinical approximation.

Worked example. Patient with BMI 32, CEAP C5, refluxing ASV, no incompetent perforators, no deep reflux, GSV 8 mm: LP = −2.097 + 0.581 + 0.468 + 0.821 + 0 + 0 + 0.334 = 0.107; p = 1/(1 + exp(−0.107)) ≈ 0.527 (52.7%).

**Adjusted associations of all candidate predictors entered into the full multivariable model (n = 10,142):**

| **Candidate predictor** | **OR (95% CI)** | **p value** | **Status in final model** |
| --- | --- | --- | --- |
| BMI ≥30 kg/m² | 1.82 (1.60–2.07) | <0.001 | Retained (2 points) |
| CEAP C4–C6 | 1.62 (1.42–1.86) | <0.001 | Retained (2 points) |
| ASV reflux* | 2.33 (2.03–2.67) | <0.001 | Retained (3 points) |
| Incompetent perforators | 2.04 (1.77–2.35) | <0.001 | Retained (3 points) |
| Deep reflux/obstruction | 1.72 (1.44–2.06) | <0.001 | Retained (2 points) |
| GSV diameter ≥7 mm | 1.41 (1.23–1.62) | <0.001 | Retained (1 point) |
| Age (per 10 years) | 1.04 (0.97–1.11) | 0.31 | Not retained |
| Female sex | 1.07 (0.94–1.21) | 0.30 | Not retained |
| Diabetes mellitus | 1.11 (0.93–1.32) | 0.25 | Not retained |
| Hypertension | 1.05 (0.92–1.20) | 0.45 | Not retained |
| Current smoking | 1.09 (0.95–1.25) | 0.22 | Not retained |
| Prior DVT (any) | 1.18 (0.96–1.45) | 0.11 | Not retained |
| Bilateral disease | 1.06 (0.94–1.20) | 0.34 | Not retained |
| Calendar era (per 6 years) | 0.94 (0.85–1.04) | 0.21 | Not retained (continuous; see Table S9) |

** ASV (anterior saphenous vein) — formerly anterior accessory saphenous vein (AASV); terminology updated to align with current consensus nomenclature.*

All 14 candidate predictors specified a priori were entered simultaneously into the full multivariable logistic regression model with cluster-robust standard errors. No selection algorithm (stepwise, forward, or backward) was used; final retention was based on a prespecified rule combining clinical relevance, statistical significance at α = 0.01 (Bonferroni-corrected), and minimum predictor count to ensure parsimony for clinical scoring. Diabetes mellitus and hypertension showed expected directional effects but did not reach the prespecified retention threshold; their potential prognostic value warrants confirmation in larger external cohorts.

## **Supplementary Table S5. Procedure-Stratified Subgroup Analysis (NEW — Revision R1)**

In response to reviewer comments R1.1, R2.1, R3.4, and R4.5, separate multivariable logistic regression models were fit within each operative subgroup. The same six retained predictors were entered into each subgroup-specific model, with cluster-robust standard errors. Performance metrics are apparent (subgroup sample sizes preclude reliable optimism correction within strata). Three primary subgroups (HL+stripping, HL alone, phlebectomy alone) are mutually exclusive and sum to the full primary cohort of 10,142. The SPJ-procedure subgroup (n = 811) is reported separately as a non-mutually-exclusive overlapping analysis (most SPJ procedures co-occur with HL+stripping for combined SFJ+SPJ disease).

| **Operative subgroup** | **n** | **Recurrences (%)** | **C-statistic (apparent)** |
| --- | --- | --- | --- |
| HL + stripping (with phlebectomies) | 7,099 | 1,755 (24.7) | 0.74 |
| HL alone (no stripping) | 1,521 | 404 (26.6) | 0.72 |
| Phlebectomy alone (no junction surgery) | 1,522 | 387 (25.4) | 0.70 |
| Subtotal — three mutually exclusive primary subgroups | 10,142 | 2,546 (25.1) | — |
| SPJ procedures (overlapping subset, mostly within HL+stripping)* | 811 | 209 (25.8) | 0.73 |
| Pooled (primary cohort, all-cause) | 10,142 | 2,546 (25.1) | 0.744 (corrected 0.744) |

** The SPJ-procedure subgroup is non-mutually-exclusive with respect to the three primary subgroups: 671 of 811 SPJ procedures (82.7%) co-occurred with HL+stripping for combined SFJ+SPJ disease and are therefore also counted within the HL+stripping subgroup; 90 (11.1%) co-occurred with HL alone; and 50 (6.2%) were isolated SPJ procedures with concurrent phlebectomy. The 209 SPJ-subgroup recurrences are accordingly a subset of the 2,546 primary-cohort recurrences and are presented here only to assess preservation of model performance in this anatomically distinct setting.*

**Subgroup-specific predictor odds ratios (95% CI):**

| **Predictor** | **HL + stripping** | **HL alone** | **SPJ procedures** | **Phlebectomy alone** |
| --- | --- | --- | --- | --- |
| ASV reflux | 2.31 (1.99–2.68) | 2.45 (1.78–3.37) | 2.18 (1.49–3.20) | 2.08 (1.51–2.86) |
| Incompetent perforators | 2.06 (1.77–2.40) | 1.94 (1.45–2.59) | 2.27 (1.59–3.24) | 1.71 (1.27–2.31) |
| BMI ≥30 kg/m² | 1.79 (1.55–2.06) | 1.91 (1.43–2.55) | 1.74 (1.21–2.50) | 1.85 (1.36–2.51) |
| Deep reflux/obstruction | 1.69 (1.40–2.04) | 1.88 (1.32–2.68) | 2.03 (1.32–3.13) | 1.51 (0.98–2.32) |
| CEAP C4–C6 | 1.59 (1.38–1.84) | 1.79 (1.34–2.39) | 1.72 (1.18–2.50) | 1.41 (1.02–1.95) |
| GSV ≥7 mm | 1.43 (1.23–1.65) | 1.32 (0.99–1.76) | —* | —† |

** In SPJ procedures, the predictor referring to GSV diameter retains the same definition (preoperative GSV diameter) when the GSV is competent and not the operated trunk; OR 1.38 (0.92–2.07). † In phlebectomy-alone procedures (no truncal involvement), GSV ≥7 mm was infrequent (n = 142) and was forced into the model for consistency; OR 1.21 (0.78–1.88).*

Predictor effect sizes were directionally concordant across all operative subgroups, with overlapping confidence intervals between strata for every predictor. The HL-alone subgroup showed a 5-year recurrence rate of 26.6% (404/1,521), modestly higher than HL+stripping (24.7%; 1,755/7,099), consistent with selective use of HL alone in patients with limited above-knee truncal reflux; nonetheless, the relative discriminative ability of the score (C-statistic 0.72) was preserved. The phlebectomy-alone subgroup (n = 1,522, recurrence 25.4%; 387/1,522) showed slightly attenuated discrimination (C-statistic 0.70), as expected when the principal anatomical risk factors (truncal reflux, junctional reflux) are by definition absent or attenuated. Numbers in this table reproduce the recurrence counts of Table 1 (HL+stripping 1,755; HL alone 404; phlebectomy alone 387; sum 2,546). These data support the use of the VVR Score across the spectrum of contemporary open-surgical approaches.

## **Supplementary Table S6. Continuous Calendar-Year Analysis with Time × Predictor Interactions (NEW — Revision R1)**

In response to reviewer comment R5.1, calendar year of surgery was modelled as a continuous variable (centred at 2015, the midpoint of the accrual period) instead of as the three categorical eras used in the main analysis. Both a main effect of calendar year and time × predictor interaction terms were tested in a single multivariable model containing the six retained predictors plus calendar year and the six interaction terms.

| **Term** | **OR (95% CI)** | **p value** | **Interpretation** |
| --- | --- | --- | --- |
| Main effect: calendar year (per year) | 0.99 (0.98–1.00) | 0.087 | Slow secular decline, not significant at α=0.05 |
| Calendar year × ASV reflux | 1.01 (0.99–1.04) | 0.31 | No interaction |
| Calendar year × Incompetent perforators | 0.98 (0.96–1.01) | 0.21 | No interaction |
| Calendar year × BMI ≥30 | 1.00 (0.98–1.02) | 0.78 | No interaction |
| Calendar year × Deep reflux/obstruction | 1.02 (0.99–1.05) | 0.24 | No interaction |
| Calendar year × CEAP C4–C6 | 0.99 (0.97–1.02) | 0.62 | No interaction |
| Calendar year × GSV ≥7 mm | 1.00 (0.98–1.03) | 0.71 | No interaction |

Model performance with continuous calendar year and full interaction terms: apparent C-statistic 0.747; optimism-corrected 0.744 — identical to the parsimonious six-variable model without calendar-year terms. The smallest interaction p value across the six time × predictor terms was 0.21 (perforators), well above any plausible threshold even without correction for multiple testing. Likelihood-ratio testing of the full model versus a reduced model excluding all seven calendar-year terms yielded p = 0.34, confirming that calendar year contributes no meaningful incremental information once the six anatomical and clinical predictors are accounted for.

Conclusion. The continuous-time analysis confirms the conclusion drawn from the categorical era-stratified model (Supplementary Table S7, analysis 7): predictor effects are stable across the 2006–2025 surgical period, and the VVR Score performance is robust to operative-era changes (cribriform fascia closure adoption, increasing concomitant perforator treatment, evolving reintervention modality mix). This temporal stability supports the score's applicability to contemporary open-surgical practice.

## **Supplementary Table S7. Summary of Sensitivity Analyses**

| **#** | **Sensitivity analysis** | **n (events)** | **C-statistic** | **Direction of effect** |
| --- | --- | --- | --- | --- |
| 1 | IPCW (all 12,480 extremities) | 12,480 (3,007*) | 0.736 (0.722) | Concordant |
| 2 | Cox regression (time-to-recurrence) | 12,480 | 0.741 (0.726) | Concordant |
| 3 | Reintervention as primary endpoint | 10,142 (1,886) | 0.730 (0.715) | Concordant |
| 4 | Excluding recurrences <6 months | 10,000 (2,404) | 0.748 (0.730) | Concordant |
| 5 | GEE (cluster correlation) | 10,142 (2,546) | 0.744 (0.728) | Concordant |
| 6 | Multiple imputation (MICE m=20) | 12,480† (3,090) | 0.741 (0.724) | Concordant |
| 7 | Era-stratified model (Era 1/2/3) | 10,142 (2,546) | 0.71–0.75 | Concordant |
| 8 | Treatment choice as covariate | 10,142 (2,546) | 0.745 (0.729) | Concordant |
| 9 | Continuous calendar-year analysis | 10,142 (2,546) | 0.744 (0.726) | Concordant (Table S9) |

** IPCW-weighted event count. † Number with at least one imputed value. Values in parentheses: optimism-corrected.*

Sensitivity analyses were prespecified and organized into three groups: (i) follow-up bias assessment (analyses 1, 2); (ii) endpoint and modelling robustness (analyses 3–7); and (iii) reviewer-requested clarifications added in revision (analyses 8–9). All analyses produced consistent results, with C-statistics ranging from 0.71 to 0.75 and directionally concordant predictor effects. Analysis 8 entered procedure type (HL alone vs HL+stripping vs phlebectomy alone) as a three-level categorical covariate; the procedure-type term was non-significant overall (likelihood-ratio p = 0.18) and effect estimates of the six retained predictors changed by ≤6% in absolute terms. Analysis 9 entered calendar year as a continuous variable (centred at 2015) and tested time × predictor interactions (see Supplementary Table S6).

## **Supplementary Table S8. Mechanism-Specific Recurrence Profiles (N = 2,245 with Documented Mechanism)**

Among 2,546 extremities with 5-year recurrence, dominant recurrence mechanism was adjudicated by duplex review in 2,245 (88.2%); 301 had insufficient documentation or coexisting mechanisms without a clear dominant pattern.

| **Dominant recurrence mechanism** | **n** | **%** |
| --- | --- | --- |
| Progression / new venous territory | 855 | 38.1% |
| Neovascularisation at SFJ/SPJ | 640 | 28.5% |
| Treated-segment recurrence — total | 489 | 21.8% |
| • Stripped GSV recanalisation | 332 | 14.8% |
| • Residual GSV stump / cribriform tributaries | 157 | 7.0% |
| Perforator-mediated recurrence | 261 | 11.6% |
| Total with classified mechanism | 2,245 | 100.0% |

A separate category — "treatment failure / immediate technical failure" (defined as residual reflux at the operated junction documented at the first postoperative duplex within 6 months) — was identified in 142 extremities; these were excluded from the recurrence numerator and described separately to avoid conflating intraoperative inadequacy with biological recurrence.

**Predictor associations by recurrence mechanism (separate multivariable logistic models):**

| **Predictor** | **Progression** | **Neovasc.** | **Treated-segment** | **Perforator** |
| --- | --- | --- | --- | --- |
| ASV reflux | 2.18*** | 2.84*** | 2.05*** | 1.61** |
| Incompetent perforators | 1.72*** | 1.34* | 1.81*** | 4.12*** |
| BMI ≥30 | 1.79*** | 1.65*** | 1.94*** | 1.71*** |
| Deep reflux/obstruction | 1.55** | 1.41* | 2.16*** | 1.62** |
| CEAP C4–C6 | 1.71*** | 1.45** | 1.52** | 1.83*** |
| GSV ≥7 mm | 1.39*** | 1.42** | 1.55** | 1.20 |

*Values are odds ratios from separate multivariable logistic models for each mechanism type. * p<0.05; ** p<0.01; *** p<0.001.*

ASV reflux was most strongly associated with neovascularisation-type recurrence; incompetent perforators were most strongly associated with perforator-mediated recurrence. BMI and CEAP showed more uniform associations across all mechanism types.

## **Supplementary Table S9. PROBAST Risk-of-Bias Assessment**

| **Domain** | **Risk of bias** | **Concerns / Mitigation** |
| --- | --- | --- |
| Participants | Low | Consecutive enrolment; clear inclusion/exclusion; large sample. |
| Predictors | Low | Predictors defined a priori; preoperative; standardized duplex protocol. |
| Outcome | Moderate | Verification/incorporation bias from management-dependent endpoint; 18.7% loss to follow-up. Reintervention sensitivity analysis (C=0.73) partly addresses this. |
| Analysis | Moderate | Single-centre derivation; no external validation. EPV adequate (424); prespecified analysis plan. |
| Overall | Low to Moderate | Outcome ascertainment and lack of external validation are the principal concerns; planned multicentre validation is the priority next step. |

PROBAST = Prediction model Risk Of Bias ASsessment Tool (Wolff RF et al., Ann Intern Med 2019;170:51–58).

Overall risk of bias: Low to Moderate. Sensitivity analyses (IPCW, Cox, reintervention as hard endpoint) mitigate but do not fully resolve the outcome ascertainment concern.

## **Supplementary Figure S1. Restricted Cubic Spline Plots**

(A) BMI and (B) GSV diameter vs. log-odds of 5-year recurrence. Red dashed lines indicate dichotomization thresholds (BMI 30 kg/m²; GSV 7 mm). Shaded areas: 95% confidence intervals. Nonlinearity was not detected (p = 0.31 for BMI; p = 0.26 for GSV diameter), supporting the use of clinical thresholds for scoring-system derivation. The near-linear relationships confirm that dichotomization at these thresholds, while not capturing the full continuous association, results in minimal information loss for the scoring system.

# **TRIPOD Checklist for Prediction Model Development and Validation**

**TRIPOD Type 1b: Development with internal validation**

Based on Collins GS et al., BMJ 2015;350:g7594 and Moons KGM et al., Ann Intern Med 2015;162:W1–W73.

| **Section/Topic** | **Item** | **Checklist Item** | **Reported on Page/Section** |
| --- | --- | --- | --- |
| Title | 1 | Identify study as developing/validating prediction model; target population and outcome | Title, p. 1 |
| Abstract | 2 | Structured summary | Abstract, p. 2 |
| Background | 3a | Medical context and rationale | Introduction ¶1–3 |
| Objectives | 3b | Study objectives including whether development, validation, or both | Introduction ¶4 |
| Source of data | 4a | Describe study design (e.g., cohort) | Methods: Study Design |
| Source of data | 4b | Key dates; start/end of accrual, follow-up | Methods: Study Design (Jan 2006–Dec 2025) |
| Participants | 5a | Key eligibility criteria | Methods: Study Population |
| Participants | 5b | Description of treatment(s) received | Methods: Surgical Protocol (NEW R1) |
| Participants | 5c | Study setting | Methods: Study Design (single centre) |
| Outcome | 6a | Clearly define outcome | Methods: Outcome Definition |
| Outcome | 6b | Actions to blind assessment of outcome | Methods: Outcome Definition (limitation discussed) |
| Predictors | 7a | Clearly define all predictors | Methods: Candidate Predictors + Duplex Protocol |
| Predictors | 7b | Actions to blind assessment of predictors | Methods: Duplex Protocol (preoperative; prior to outcome) |
| Sample size | 8 | How sample size was determined | Methods: Sample Size (EPV = 424) |
| Missing data | 9 | Handling of missing data | Methods: Missing Data (complete-case + MICE) |
| Statistical analysis | 10a | Describe how predictors were handled | Methods: Statistical Analysis |
| Statistical analysis | 10b | Type of model, selection, interaction assessment | Methods: Statistical Analysis |
| Statistical analysis | 10c | Internal validation method | Methods: Statistical Analysis (cluster bootstrap, 500 reps) |
| Statistical analysis | 10d | Performance measures (discrimination, calibration) | Methods + Table 6 |
| Statistical analysis | 10e | Describe any model updating | N/A (development study) |
| Risk groups | 11 | Meaningful risk groups | Results: VVR Score; Table 5 |
| Participants | 13a | Flow of participants (diagram) | Results: Study Population; Figure 1 |
| Participants | 13b | Baseline characteristics by outcome | Table 1 |
| Participants | 13c | For validation: show distribution of predictors | N/A (Type 1b) |
| Model development | 14a | Specify unadjusted and adjusted associations | Tables 2–3 + Suppl Table S1 |
| Model development | 14b | Full prediction model | Suppl. Table S1 (full equation + non-retained) |
| Model specification | 15a | Discrimination, calibration | Results: Model Performance; Table 6; Figures 2–3 |
| Model specification | 15b | Performance for key subgroups | Suppl. Tables S5 + S8 (NEW R1) |
| Results | 16 | Other analyses (e.g., sensitivity) | Results: Sensitivity Analyses; Suppl. Tables S5, S8, S9 |
| Discussion | 18 | Discuss limitations | Discussion: Limitations (PROBAST: Suppl. Table S6) |
| Interpretation | 19a | Discuss results with reference to objectives | Discussion ¶1–5 |
| Interpretation | 19b | Overall interpretation of results | Discussion ¶1; Conclusions |
| Implications | 20 | Potential clinical use and implications | Discussion: Clinical Applications |
| Supplementary | 21 | Provide information about full model | Suppl. Table S1 (full equation + non-retained) |
| Funding | 22 | Give source of funding | Funding (none) |

*All items addressed. Items marked "N/A" are not applicable to TRIPOD Type 1b (development with internal validation only).*
